# Community Case Management of Fever Due to Malaria and Pneumonia in Children Under Five in Zambia: A Cluster Randomized Controlled Trial

**DOI:** 10.1371/journal.pmed.1000340

**Published:** 2010-09-21

**Authors:** Kojo Yeboah-Antwi, Portipher Pilingana, William B. Macleod, Katherine Semrau, Kazungu Siazeele, Penelope Kalesha, Busiku Hamainza, Phil Seidenberg, Arthur Mazimba, Lora Sabin, Karen Kamholz, Donald M. Thea, Davidson H. Hamer

**Affiliations:** 1 Center for Global Health and Development, Boston University School of Public Health, Boston, Massachusetts, United States of America; 2 Chikankata Mission Hospital, Chikankata, Southern Province, Zambia; 3 Child Health Unit, Ministry of Health, Lusaka, Zambia; 4 National Malaria Control Center, Ministry of Health, Lusaka, Zambia; 5 Center for International Health and Development-Zambia, Lusaka, Zambia; 6 Department of Pediatrics, Boston University School of Medicine, Boston, Massachusetts, United States of America; 7 Section of Infectious Diseases, Department of Medicine, Boston University School of Medicine, Boston, Massachusetts, United States of America; London School of Hygiene and Tropical Medicine, United Kingdom

## Abstract

**Background:**

Pneumonia and malaria, two of the leading causes of morbidity and mortality among children under five in Zambia, often have overlapping clinical manifestations. Zambia is piloting the use of artemether-lumefantrine (AL) by community health workers (CHWs) to treat uncomplicated malaria. Valid concerns about potential overuse of AL could be addressed by the use of malaria rapid diagnostics employed at the community level. Currently, CHWs in Zambia evaluate and treat children with suspected malaria in rural areas, but they refer children with suspected pneumonia to the nearest health facility. This study was designed to assess the effectiveness and feasibility of using CHWs to manage nonsevere pneumonia and uncomplicated malaria with the aid of rapid diagnostic tests (RDTs).

**Methods and Findings:**

Community health posts staffed by CHWs were matched and randomly allocated to intervention and control arms. Children between the ages of 6 months and 5 years were managed according to the study protocol, as follows. Intervention CHWs performed RDTs, treated test-positive children with AL, and treated those with nonsevere pneumonia (increased respiratory rate) with amoxicillin. Control CHWs did not perform RDTs, treated all febrile children with AL, and referred those with signs of pneumonia to the health facility, as per Ministry of Health policy. The primary outcomes were the use of AL in children with fever and early and appropriate treatment with antibiotics for nonsevere pneumonia. A total of 3,125 children with fever and/or difficult/fast breathing were managed over a 12-month period. In the intervention arm, 27.5% (265/963) of children with fever received AL compared to 99.1% (2066/2084) of control children (risk ratio 0.23, 95% confidence interval 0.14–0.38). For children classified with nonsevere pneumonia, 68.2% (247/362) in the intervention arm and 13.3% (22/203) in the control arm received early and appropriate treatment (risk ratio 5.32, 95% confidence interval 2.19–8.94). There were two deaths in the intervention and one in the control arm.

**Conclusions:**

The potential for CHWs to use RDTs, AL, and amoxicillin to manage both malaria and pneumonia at the community level is promising and might reduce overuse of AL, as well as provide early and appropriate treatment to children with nonsevere pneumonia.

**Trial registration:**

ClinicalTrials.gov NCT00513500

*Please see later in the article for the Editors' Summary*

## Abstract 2

In a cluster randomized trial, Kojo Yeboah-Antwi and colleagues find that integrated management of malaria and pneumonia in children under five by community health workers is both feasible and effective.

## Editors' Summary

**Background:**

Every year, about 11 million children die before their fifth birthday. Most of these deaths are in developing countries and most are due to a handful of causes—pneumonia (lung inflammation usually caused by an infection), malaria (a parasitic disease spread by mosquitoes), measles, diarrhea, and birth-related problems. In sub-Saharan Africa, pneumonia and malaria alone are responsible for nearly a third of deaths in young children. Both these diseases can be treated if caught early—pneumonia with antibiotics such as amoxicillin and malaria with artemisinin-based combination therapy (ACT), a treatment that contains several powerful antimalarial drugs. Unfortunately, parents in rural areas in sub-Saharan Africa rarely have easy access to health facilities and sick children are often treated at home by community health workers (CHWs, individuals with some medical training who provide basic health care to their communities), drug sellers, and traditional healers. This situation means that ongoing global efforts to reduce child mortality will require innovative community level interventions if they are to succeed.

**Why Was This Study Done?:**

One community level intervention that the World Health Organization (WHO) and the United Nations Children's Fund (UNICEF) recently recommended is integrated management of malaria and pneumonia in countries where these diseases are major childhood killers. One such country is Zambia. In rural areas of Zambia, CHWs treat suspected cases of uncomplicated (mild) malaria with artemether-lumefantrine (AL, an ACT) or with sulfadoxine-pyrimethamine (a non-ACT antimalarial drug combination) and refer children with suspected pneumonia to the nearest health facility. However, because uncomplicated malaria and pneumonia both cause fever, many children are treated inappropriately. This misdiagnosis is worrying because giving antimalarial drugs to children with pneumonia delays their treatment with more appropriate drugs and increases the risk of drug-resistant malaria emerging. The use of rapid diagnostic tests (RDTs) for malaria might be one way to improve the treatment of malaria and pneumonia by CHWs in Zambia. Here, the researchers investigate the feasibility and effectiveness of this approach in a cluster randomized controlled trial, a study that compares the outcomes of groups (clusters) of patients randomly allocated to different interventions.

**What Did the Researchers Do and Find?:**

The researchers randomly allocated 31 community health posts (fixed locations where CHWs provide medical services to several villages) to the study's intervention and control arms. CHWs in the intervention arm did RDTs for malaria on all the children under 5 years old who presented with fever and/or difficult or fast breathing (symptoms of pneumonia), treated test-positive children with AL, and treated those with nonsevere pneumonia (an increased breathing rate) with amoxicillin. CHWs in the control arm did not use RDTs but treated all children with fever with AL and referred those with signs of pneumonia to the nearest health facility. About 3,000 children with fever were treated during the 12-month study. 99.1% of the children in the control arm received AL compared with 27.5% of the children in the intervention arm, a 4-fold reduction in treatment for malaria. Importantly, the CHWs in the intervention arm adhered to treatment guidelines and did not give AL to children with negative RDT results. Of the children classified with nonsevere pneumonia, 13.3% of those in the control arm received early and appropriate treatment with amoxicillin compared to 68.2% of those in the intervention arm, a 5-fold increase in the timely treatment for pneumonia.

**What Do These Findings Mean?:**

These findings indicate that CHWs in Zambia are capable of using RDTs, AL, and amoxicillin to manage malaria and pneumonia. They show that the intervention tested in this study has the potential to reduce the overuse of AL and to provide early and appropriate treatment for nonsevere pneumonia. Although this approach needs to be tested in other settings, these findings suggest that the use of CHWs might be a feasible and effective way to provide integrated management of pneumonia and malaria at the community level in developing countries. Importantly, these results also support the evaluation of the treatment by CHWs of other major childhood diseases and raise the possibility of saving the lives of many children in sub-Saharan Africa and other developing regions of the world through community level interventions.

**Additional Information:**

Please access these Web sites via the online version of this summary at http://dx.doi.org/10.1371/journal.pmed.1000340.

- WHO provides information on malaria, on rapid diagnostic tests for malaria, on artemisinin-combination therapy, and on global child mortality and efforts to reduce it (in several languages); WHO also provides a country health profile for Zambia
- The US Centers for Disease Control and Prevention provide information on malaria (in English and Spanish)
- Kidshealth, a resource maintained by the not-for-profit Nemours Foundation (a not-for-profit organization for children's health), provides information for parents on pneumonia (in English and Spanish)
- MedlinePlus provides links to additional information on malaria and on pneumonia (in English and Spanish)
- More information about the Zambia Integrated Management of Malaria and Pneumonia Study is available

## Introduction

Pneumonia and malaria are major causes of morbidity and mortality in children under five in sub-Saharan Africa [1],[2]. In many rural areas in developing countries, health facilities are not readily accessible to much of the population [3],[4], and the health needs of large numbers of sick children are met through the informal sector, including community health workers (CHWs), drug sellers, and traditional healers [5]. Since access to health facilities is limited and most children die at home [5], new and innovative approaches to reducing childhood mortality will require interventions implemented at the community level. The World Health Organization (WHO) and the United Nations Children Fund (UNICEF) now recommend that where malaria and pneumonia are major killers, their treatment should be integrated in community case management activities [6].

Many pneumonia deaths could be prevented through early, appropriate and low-cost community-based treatment [7],[8]. Training CHWs in the management of acute lower respiratory tract infections has been shown to be feasible and effective [9],[10]. Similarly, several studies have shown that CHWs can be trained to provide effective malaria case management at the community level [11],[12].

Following WHO recommendations, artemisinin-based combination therapy (ACT) has been introduced as the first line treatment for uncomplicated malaria in much of sub-Saharan Africa [13],[14]. The use of rapid diagnostic tests (RDTs) for guiding malaria treatment has been found to offer a practical solution to the challenge of malaria diagnostics in Africa [15],[16]. As a result, RDTs are increasingly being utilized to improve malaria case management and reduce unnecessary ACT use [17]. Because the overlap of symptoms between malaria and pneumonia in children makes differential diagnosis in the absence of diagnostic equipment difficult [18],[19], RDT use by CHWs presents a remarkable opportunity to improve the diagnosis of malaria and pneumonia at the community level. The use of RDTs by CHWs has been found to be potentially effective and feasible [20]–[23]. Although ACTs and RDTs are now available at health facilities in Zambia, they have not been fully deployed at the community level.

Few studies have evaluated the integrated management of fever due to pneumonia and malaria by CHWs in children [24],[25], and the strategy of having CHWs dispense ACT has not been carefully evaluated. In addition, the potential benefit(s) of RDTs in improving malaria diagnosis before treatment with ACT by CHWs remain unknown. The objective of this study was, therefore, to assess the effectiveness and feasibility of using CHWs to manage pneumonia and malaria in children with the aid of RDTs per our protocol (Text S1).

## Methods

We report here, using the Consort checklist (Text S2), a cluster randomized controlled trial that compared two models for community-based management of malaria and/or nonsevere pneumonia in children in rural Zambia. A cluster design was used instead of individual randomization because it was socially and culturally inappropriate for a CHW to give one patient treatment for nonsevere pneumonia and to refer the next patient to a health facility. Baseline and poststudy household surveys were conducted to assess changes in health-seeking behavior.

### Study Area and Participants

The study was conducted in the Chikankata Mission Hospital catchment area, a geographic area with an estimated population of 70,000 [26] extending across parts of Siavonga and Mazabuka Districts in Zambia's Southern Province. The Siavonga area is predominantly plain and referred to as “the valley”; and the Mazabuka area is hilly and referred to as “the plateau.” Malaria, malnutrition, pneumonia, and diarrhea are the leading causes of morbidity and death in children under five [27],[28]. Transmission of malaria is hyperendemic and highest in the rainy season from November to April [29]. The study area has poor road networks and is served by the mission hospital and five official Zambian Ministry of Health rural health centers, of which only one has a full complement of staff (clinical officer, environmental health technician, and midwife). Most sick children are seen by CHWs who work in a fixed location called the community health post, which serves a number of villages. At the time of study initiation, CHWs did not use ACT, RDTs, or amoxicillin. Instead they would treat malaria with sulfadoxine-pyrimethamine and refer suspected pneumonia cases to the nearest health facility. They also routinely managed children presenting with diarrhea and dehydration with oral rehydration therapy. Two to eight health posts are situated within a health center catchment area. There was no formalized incentive package for the CHWs, but the Chikankata Mission Hospital provided occasional incentives (i.e., bicycles, umbrella, and stationery) when resources were available.

Children aged between 6 mo and 5 y who presented to a CHW at a health post with fever and/or cough/difficult breathing/fast breathing were eligible for participation. Exclusion criteria included age below 6 mo or above 5 y, signs or symptoms of severe illness, or known sensitivity to the study medications.

### The Intervention

#### Training

All study CHWs had previously undergone 6 wk training before becoming community health workers. As part of the study, they participated in an additional 5-d training workshop using modifications of nationally developed training manuals. Members from both the Mazabuka and Siavonga District Health Management Teams (DHMT), study staff, and the principal investigator (KYA) led the training workshop. Training team members were experienced in training Integrated Management of Childhood Illness (IMCI) skills.

The workshop had two main parts. In one part, which lasted 4 d, both intervention and control CHWs were trained, using the CHW training manual (Text S3), to classify and manage children with pneumonia and/or malaria, and to manage stocks of drugs and supplies. The training of the CHWs was highly interactive and included a variety of methods including lectures, discussion, role play, demonstrations, case studies, and supervised clinical practice at the hospital. The training emphasized community-based integrated management of febrile children including basic clinical history taking, physical examination skills, counseling of caregivers, and recognition of signs of severe illness requiring referral. A major focus was training CHWs in the use of simplified treatment algorithms developed to aid classification and treatment of malaria and pneumonia, with separate algorithms for the intervention and the control CHWs. Each algorithm had three sections; section A was used for children who presented with fever plus cough and/or shortness of breath (fast breathing, difficult breathing); section B for children who presented with cough and/or shortness of breath (fast breathing, difficult breathing) but without fever; and section C for children who had fever alone, without cough and/or shortness of breath. The identification of danger signs was an important focus of the training, to help ensure that the child, if in danger, was immediately referred to the nearest health center. The CHWs were also trained in the use of simple dosing guidelines based on weight, if available, or age for artemether-lumefantrine (AL). As part of this component of the training, both groups of CHWs were trained on how to manage the drug supplies, including proper documentation of patient complaints, their physical examination, and medications administered to the child.

The second part of the workshop involved additional training for the intervention CHWs only. The CHW RDT training manual (Text S4) was used for this half-day session, which focused on performing and interpreting RDTs, with the aid of RDT interpretation guides. The proper interpretation of RDT results was emphasized, including how to react to both positive and negative results. As part of the RDT training, intervention CHWs were trained in infection control measures such as aseptic technique, proper disposal of hazardous biological waste, and avoidance of lancet injuries. The intervention CHWs also received supplemental training in amoxicillin dosing and documentation of RDT results.

After completion of training, the instructors assessed the competency of all CHWs to count respiratory rate and follow treatment algorithms, and, for the intervention CHWs, the proper performance and interpretation of RDTs. One month after initial training, the training team completed a follow-up skills assessment to ensure that CHWs retained the necessary skills. All study CHWs completed an additional 2-d refresher course 6 mo after the initial training.

Specialized data collectors were recruited and trained in study procedures, research ethics, informed consent protocols, and the use of data collection instruments.

#### Patient management and follow-up

Intervention CHWs performed RDTs on children with reported fever and counted the respiratory rate of children with cough and/or difficult/fast breathing using a respiratory timer. They classified children with positive RDT results and normal respiratory rate as malaria and treated with AL and an antipyretic (acetaminophen). Children with positive RDT results and high respiratory rate (≥50 breaths per minute in children <12 mo; ≥40 breaths per minute in children ≥12 mo) were classified as having both malaria and nonsevere pneumonia and were treated with AL, amoxicillin, and acetaminophen. Children with negative RDT results and high respiratory rate were classified as nonsevere pneumonia and treated with amoxicillin. Children with negative RDT results and normal respiratory rate were classified as “fever with negative RDT” and treated with acetaminophen. Control CHWs did not perform RDTs but counted the respiratory rate with a timer. They classified children with reported fever and normal respiratory rate as malaria and treated them with AL and acetaminophen. Children with reported fever and high respiratory rate were classified as having both malaria and nonsevere pneumonia and were treated with AL and acetaminophen and then referred to the nearest health facility. Children with no reported fever but high respiratory rate were classified as nonsevere pneumonia and referred to the nearest health center for management. In both arms, the CHWs weighed the children and measured the temperature of those with reported fever using digital thermometers.

The CHWs completed a baseline and identification form for every child managed according to the study protocol. The form documented presenting complaints, basic examination findings, RDT results (for intervention sites), illness classification, and treatment provided. A data collector made contact with a CHW every other day to collect the baseline and identification forms of newly managed children. The data collector then visited the patient at home on day 5–7 after the initial visit to the CHW, obtained informed consent, interviewed the child's caregiver, and performed a basic examination of the child, including measuring and recording temperature and respiratory rate. Data collected and documented on the follow-up visit case report form included treatment and counselling/advice given by the CHW (as reported by the caregiver), referrals, self-report of adherence to medication, alternative treatment after the visit to the CHW, and current conditions and complaints.

#### Study supplies

CHWs in the intervention arm were supplied with RDTs, AL, and amoxicillin, while the control CHWs were supplied with AL only. All CHWs were given acetaminophen. The drugs and RDTs were collected from the District Health Management Teams and provided to CHWs monthly. The RDT used was ICT Malaria Pf (ICT Diagnostics,), which has high sensitivity (95%–98%) and specificity (75%–80%) in field settings and is increasingly being recommended for field use [15],[30],[31]. The test is based on the detection of *Plasmodium falciparum* histidine-rich protein 2 in whole blood. Ensuring quality of the RDT kits was outside the scope of this study. However, the performance assessment conducted at the health center assessed how the CHWs performed and interpreted the results of the RDTs. Constant review of CHWs' records on RDT use and feedback also proved useful. All RDTs procured by the Ministry of Health undergo lot testing at the central level before distribution to districts. Any lot found to be of poor quality is not distributed. The formulation of AL used was Coartem (Novartis Pharmaceuticals Corporation for Novartis Pharma AG) or Lumet (Cipla LTD). Both formulations were supplied in packs of six or 12 tablets (20 mg artemether, 120 mg lumefantrine). The amoxicillin (Sparsh BIO-TECH PVT LTD) was prepackaged into prescription envelopes in two different doses of eight tablets (250 mg) (one half-tablet three times per day for 5 d) for younger children (weighing <10 kg or aged 6–11 mo) or 15 tablets (one tablet three times per day for 5 d) for older children (weighing ≥10 kg or aged 12 mo to 5 y). All CHWs were also supplied with digital thermometers, respiratory counters, and weighing scales.

#### Supervision and performance assessment

CHWs made monthly visits to health centers with their registers and record books. During these visits, the head nurse of each health center checked the registers and records, observed the work of the CHW, and assessed their performance, including how to perform and interpret the results of RDTs, at least once every 3 mo. Prior to the study, a few CHWs were already visiting health centers, but the project institutionalized this practice because it was both acceptable to the CHWs and health center staff and clearly beneficial to both groups. The CHWs were provided with bicycles to enable them to make these visits. Refresher training was carried out after 6 mo of implementation to maintain CHWs' skills.

### Study Outcomes

There were two primary outcomes: (1) the proportion of children presenting with fever who received AL and (2) the proportion of children classified as nonsevere pneumonia that received early and appropriate treatment. Early and appropriate treatment was defined as receiving 13–15 doses of amoxicillin over 5 d and receiving the first dose within 24–48 h of onset of first symptom. The main secondary outcome was the proportion of children who experienced treatment failure. Treatment failure was defined as the presence of any of the following: (1) fever, fast breathing, or difficulty in breathing as reported by care giver, temperature ≥37.5°C or high respiratory rate; (2) lower chest in-drawings or serious illness; (3) received additional antibiotics; (4) received additional antimalarials; (5) hospitalization; and (6) death. Treatment failure was assessed at the 5–7 day follow-up visit. The first primary outcome was measured from the baseline form, while the second primary and secondary outcomes were measured from the day 5–7 form.

### Sample Size

For the first primary outcome, we assumed an intracluster coefficient (ICC) of 0.25, a minimum of 33 patients per cluster, and 80% power to detect a difference of 20% in the proportions between the two arms with a two-sided alpha of 0.05. With anticipated drop outs of 15%, we needed to enrol at least 1,518 patients with fever in the arms combined. For the second primary outcome, we again assumed an ICC of 0.25, a minimum of 12 patients per cluster, and 80% power with a two-sided alpha of 0.05. With anticipated drop outs at 15%, we needed to enrol a minimum of 524 patients classified as nonsevere pneumonia in the arms combined. We used a methodology suggested by Hayes and colleagues in the sample size calculation [32].

### Randomization

For logistical reasons, community health posts situated more than 15 km from a rural health center or Chikankata Mission Hospital were excluded from the study. Community health posts that did not have a CHW at the start of the study were also excluded. Functional community health posts located in a health center catchment area were matched in pairs according to the distance from the health facility. A random number generator was used to assign one post in the pair to the control arm, while the matched post was assigned to the intervention arm. One health center catchment area had five community health posts so one post did not have a matched pair and was assigned to the control arm. We did not match according to the size or population of the community health post catchment area because this information was not available at the start of the study.

### Data Management and Analysis

Data collectors submitted their case report forms twice a week to the data supervisor who checked them for completeness. The data were double-entered into CSPro 3.3 (US Census Bureau); consistency and validation checks were conducted. Analyses were completed using SAS v 9.1.3 (SAS Institute). We compared crude proportions of the study outcomes in the two groups using the Mantel-Haenszel chi-square test with clusters as strata to account for cluster randomization. Risk ratios (RRs) and 95% confidence intervals (CIs) were calculated using generalized estimating equations with an exchangeable correlation matrix accounting for the clustering [33]. We adjusted for baseline symptoms and adjusted RRs are presented. We made inferences about treatment effects at the individual level rather than cluster level. Analyses were based on an intent-to-treat basis.

### Household Surveys and Other Data Collection

Baseline and poststudy household surveys were conducted with women aged 15–45 y who had at least one child aged 5 y or less and resided in the study area. Two villages per community health post area were randomly selected and the same villages were used in both baseline and poststudy surveys. Information collected included: health-seeking practices, recent child morbidity and mortality, adherence to treatment, utilization and acceptability of CHW services, and knowledge of signs and symptoms of severe childhood illnesses. Routine health information (patients seen and per category, availability of drugs and RDTs, referrals) were collected from the community health posts and rural health centers. Baseline data including demography, training, and support were collected from the CHWs.

### Ethical Clearance

Ethical approval was obtained from Boston University's Institutional Review Board (IRB) and the Research Ethics Committee of the University of Zambia. The study was also approved by the Zambian Ministry of Health and the Siavonga and Mazabuka District Health Management Teams. Approval was sought and obtained from community leaders and village headmen. Informed consent was obtained from the CHWs prior to their training in order to collect baseline information and to review their work during the course of the study. We obtained informed consent from caregivers at the time of the follow-up visits rather than at the time they sought care from the CHWs. We used this approach, which was approved by the Boston University IRB, because the care that was provided was considered to be standard care at the community health post (control) and rural health center (intervention); and the caregivers were free to reject all or part of the treatment. The caregiver consent was performed in order to allow us to collect information to assess the outcome of the treatment received by the child.

A data safety monitoring board reviewed the primary outcomes and adverse events after 6 mo of implementation and recommended the continuation of the study. The trial was registered online at http://register.clinicaltrial.gov with registration number NCT00513500.

## Results

**Figure 1:**
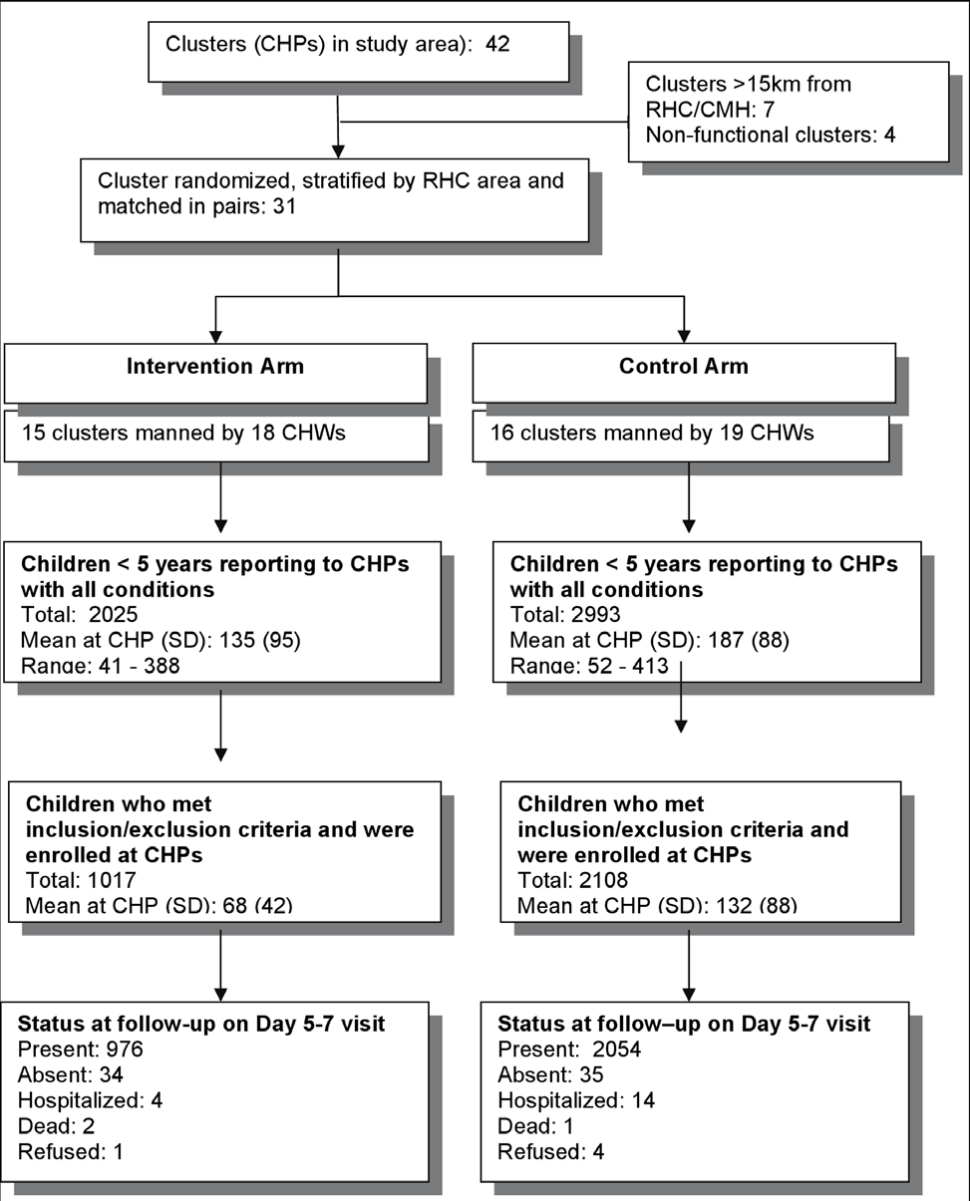
Study profile.

Participants were enrolled between December 2007 and November 2008. 31 of the 42 community health posts in the study area, which were functional and within 15 km of a health center, were randomized into intervention and control arms. A total of 3,125 children who met the eligibility criteria were enrolled, including 1,017 in the intervention arm and 2,108 in the control arm (Figure 1). Loss to follow-up was 3.3% in the intervention arm and 1.7% in the control arm.

### Baseline Characteristics

The mean distance between a community health post and the nearest health center was similar in the intervention and control arms (Table 1). Baseline characteristics of the intervention and control CHWs were also comparable. The average age of CHWs was 40.3 y in the intervention arm and 39.0 y in the control arm; most CHWs in both arms were male. The notable difference was the reported proportion of time spent on community health work. More CHWs in the control arm considered themselves full time compared to those in the intervention arm, although this difference was not significant. Almost all of the CHWs indicated that providing useful services to their communities was the key motivation for continuing to work as a CHW.

**Table 1:** Characteristics of community health workers and community health posts.

Characteristic	Intervention	Control	RR (95% CI)
Mean distance in kilometers of community health post from health center (range)	9.2 (1–15)	9.3 (3–15)	—
Proportion of male CHWs	83.3%	89.5%	0.78 (0.35–1.75)
Average age of CHWs in years (range)	40.3 (26–53)	40.0 (27–55)	—
Proportion of CHWs with secondary education	72.2%	64.4%	1.10 (0.52–2.34)
Proportion CHWs considered as full time	5.6%	26.3%	0.30 (0.05–1.87)
Mean years of practice as CHW	10.2 (1–26)	7.3 (1–22)	
Proportion of CHWs who received refresher training less than 1 y before the start of the study	55.6%	52.6%	1.06 (0.54–2.07)
Proportion of CHWs who received supervisory visit from health center staff within 3 mo before the start of the study	55.6%	52.6%	1.06 (0.54–2.07)

The baseline characteristics of enrolled children are presented in Table 2. The mean age was a little under 24 mo and there was a slight male predominance in both groups. With the exception of immunization status, which was lower in the intervention group, all basic characteristics across the two arms were comparable. Insecticide-treated net (ITN) use was quite high (70.1%) in this population and a substantial proportion (30.1%) of the children were malnourished.

**Table 2:** Baseline characteristics of children managed by community health workers.

Characteristic	Intervention (*n* = 1017)	Control (*n* = 2,108)	RR (95% CI)
Sex (female)	47.6%	48.8%	0.97 (0.87–1.07)
Mean age in mo (SD)	22.7 (14.1)	23.8 (14.8)	—
Proportion of children malnourished (WAZ score <−2.0)	28.1%	30.3%	0.93 (0.83–1.04)
Mother's education: proportion with secondary education	9.1%	8.1%	1.09 (0.92–1.30)
Mother's occupation: proportion who are housewife/farmer	94.6%	93.3%	1.17 (0.93–1.47)
Households with six or fewer persons	64.2%	62.6%	1.03 (0.92–1.14)
Proportion with up-to-date immunization^a^	59.5%	67.5%	0.79 (0.72–0.88)
Proportion slept under insecticide-treated nets the previous night	71.3%	69.5%	1.06 (0.95–1.19)

a Up-to-date immunization, received all immunizations for age as per national guidelines.

SD, standard deviation; WAZ, weight for age Z-score.

### Presenting Complaints and Disease Classification

In both arms, a majority of the children presented with a history of fever, and approximately half of these children had a measured temperature of ≥37.5°C (Table 3). A significantly higher proportion of children in the intervention arm complained of fast breathing (RR 2.45, 95% CI 2.24–2.68) or difficult breathing (RR 1.80, 95% CI 1.60–2.02). Among the subset of children classified as having pneumonia, 28.2% (102/362) in the intervention arm were classified as having both malaria and pneumonia compared to 87.2% (177/203) in the control arm (RR 0.32, 95% CI 0.27–0.38). CHW adherence to the study algorithm for classification of malaria and/or pneumonia was very high in both arms (e.g., >95%).

**Table 3:** Presenting complaints and signs.

Complaint or Sign	Intervention (*n* = 1,017)	Control (*n* = 2,108)	RR (95% CI)
Fever	94.7%	98.9%	0.45 (0.39–0.53)
Fever with temperature ≥37.5°C	45.5%	50.8%	0.87 (0.78–0.96)
Cough	67.8%	63.3%	1.15 (1.03–1.28)
Difficult breathing	16.8%	6.9%	1.80 (1.60–2.02)
Fast breathing by history	35.8%	10.2%	2.45 (2.24–2.68)
Fast breathing based on respiratory rate counted by the community health worker	37.6%	9.7%	2.61 (2.31–2.85)
Caregiver visited community health post on the same day as the first symptom onset	12.1%	10.1%	1.14 (0.98–1.33)

### RDT Results and Treatment for Malaria

Of the 975 children in the intervention arm who presented with a history of fever and had RDTs performed, 27.8% had a positive RDT result. RDT positivity varied by geographical area and rural health center catchment area, and was higher in children seen at community health posts located in the “valley” (Table 4). Of the 975 children with reported fever, 460 had a measured temperature of ≥37.5°C. The proportion of children with a positive RDT in this subgroup was 28.5%; hence there was no difference in the RDT positivity rate whether the child had reported fever or had a measured temperature of ≥37.5°C.

**Table 4:** Proportion of children with positive rapid diagnostic tests.

Children	*n* RDTs done	*n* RDT positives	Percent Positive
All children	975	271	27.8
Children with temperature ≥37.5°C	460	131	28.5
**Geographic Location**			
Valley (Siavonga)	487	219	45.0
Plateau (Mazabuka)	488	52	10.7
**Community Health Post**			
Sianyoolo	239	88	36.8
Chaanga	248	131	52.8
Chikankata	66	1	1.5
Nameembo	88	6	6.8
Nadezwe	134	6	4.5
Chikombola	200	39	19.5

The proportion of children presenting with a history of fever that received AL in the intervention arm was 27.5% compared to 99.1% in the control arm (RR 0.23, 95% CI 0.14–0.38). Only three of the 704 children with negative RDT results were given AL by the CHW. Caregivers of five children with negative RDT results sought and received antimalarials from other sources after the CHW did not provide them.

### Early and Appropriate Treatment for Pneumonia

Of the children classified as nonsevere pneumonia in the intervention arm, 78.8% sought treatment (consulted a CHW) within 24–48 h of onset of first symptom and 68.2% received early and appropriate treatment. In the control arm, 75.4% of children classified as nonsevere pneumonia sought treatment within 24–48 h of onset of first symptom and only 13.3% received early and appropriate treatment. While there was no significant difference between the two arms in the proportions of children who sought treatment within 24–48 h of onset of first symptom (RR 1.06, 95% CI 0.91–1.23), the difference in the proportions that received early and appropriate treatment for nonsevere pneumonia was significant (RR 5.32, 95% CI 2.19–8.94). Several factors, including age of the child, presenting complaints, maternal age and education, were examined either as promoters of or barriers to early and appropriate treatment. Children ≤11 mo tended to be less likely to receive early and appropriate treatment compared to older children (RR 0.84, 95% CI 0.69–1.02). Children of mothers with primary or secondary education tended to receive early and appropriate treatment compared to children of women without any education (RR 1.18, 95% CI 0.97–1.45). However, maternal age and type of presenting complaint did not influence the achievement of early and appropriate treatment.

### Treatment Failure

There was no difference in the overall treatment failure rates among patients enrolled in the intervention (9.3%) and control (10.0%) arms (Table 5). Similarly, there was no significant difference between arms in treatment failure rate among children classified as having malaria (Table 6). However, children in the intervention group who were classified as having nonsevere pneumonia were significantly less likely to experience treatment failure (RR 0.44, 95% CI 0.21–0.92). The most common reasons for treatment failure in both arms were persistent fever and fast/difficult breathing at follow up. Hospitalization was also an important reason for treatment failure in the control arm (Table 7). Two patients in the intervention arm and one in the control arm died. The final outcomes of hospitalized patients were not determined nor were verbal autopsies performed to ascertain the cause of death.

**Table 5:** Treatment failure for all patients.

Variable	Intervention	Control	RR^a^ (95% CI)
Treatment failure at day 5–7	95/1,017 (9.3%)	211/2,108 (10.0%)	0.68 (0.39–1.19)
Persistent fever, fast/difficult breathing at follow-up	73/975 (7.5%)	159/2,052 (7.7%)	0.74 (0.42–1.29)
Lower chest in-drawing at follow-up	1/973 (0.1%)	9/2,052 (0.4%)	0.17 (0.01–2.11)
Received additional antibiotics	13/975 (1.3%)	25/2,054 (1.2%)	0.94(0.19–4.79)
Received additional antimalarials	4/975 (0.4%)	8/2,054 (0.4%)	1.24 (0.41–3.57)
Hospitalization	4/1,017 (0.4%)	14/2,108 (0.7%)	0.25 (0.04–1.50)
Death	2/1,017 (0.2%)	1/2,108 (0%)	—

a Adjusted for baseline fast breathing and fever.

**Table 6:** Treatment failure for patients classified as malaria.

Variable	Intervention	Control	RR^a^ (95% CI)
Treatment failure at day 5–7	20/272 (7.4%)	207/2,082 (9.9%)	0.68 (0.38–1.19)
Persistent fever, fast/difficult breathing at follow up	17/255 (6.7%)	155/2,026 (7.7%)	0.86 (0.51–1.45)
Lower chest in-drawing at follow-up	0/253 (0%)	9/2,026 (0.4%)	
Received additional antibiotics	2/255 (0.8%)	25/2,028 (1.2%)	0.58 (0.09–3.96)
Received additional antimalarials	1/255 (0.4%)	8/2,025 (0.4%)	—
Hospitalization	0/272 (0%)	14/2,082 (0.7%)	—
Death	0/272 (0%)	1/2,082 (0%)	—

a Adjusted for baseline fever.

**Table 7:** Treatment failure for patients classified as pneumonia.

Variable	Intervention	Control	RR^a^ (95% CI)
Treatment failure day 5–7	41/362 (11.3%)	41/203 (20.2%)	0.44 (0.21–0.93)
Persistent fever, fast/difficult breathing at follow up	36/344 (10.5%)	32/193 (16.6%)	0.50 (0.22–1.17)
Lower chest in-drawing on presentation at follow-up	0/344 (0%)	2/193 (1.0%)	
Received additional antibiotics	3/344 (0.9%)	1/193 (0.5%)	1.71 (0.18–16.2)
Received additional antimalarials	1/344 (0.3%)	0/193 (0)	
Hospitalization	2/362 (0.6%)	7/203 (3.4%)	0.13 (0.02–0.75)
Death	1/362 (0.3%)	0/203 (0%)	

a Adjusted for baseline fast breathing.

Among children classified as having nonsevere pneumonia in the control arm, 14 (6.8%) were not referred by the CHWs as per standard of care and training. Of those who were referred to a health center, 22% did not comply with the referral. The major reason for noncompliance was that the caregiver did not believe the child's illness was serious enough to warrant referral, particularly when the child had been given treatment for malaria.

### Health-Seeking Practices

During the household surveys, 439 and 441 women were interviewed in the baseline and postintervention surveys, respectively. Table 8 shows the changes in health-seeking practices that occurred between the beginning and end of the 1-y study period. There was a significant shift in where sick children sought care between the preintervention (baseline) and the postintervention surveys in both arms. In the postintervention survey, the proportion that sought care from CHWs increased while there was a corresponding decrease in the proportion that sought care at the rural health centers or resorted to home care. However, for children with fast/difficult breathing, the same shift only occurred in the intervention arm. The most common reasons for not seeking care with a CHW were unavailability of the CHW (45%), sickness perceived to be too severe for the CHW to handle (12%), and being nearer to the rural health center than to the community health post (10%).

**Table 8:** Proportion of children seeking care for all illnesses and fast breathing during the baseline and poststudy household surveys.

Source of care	Intervention Baseline	Intervention Poststudy	Control Baseline	Control Poststudy
**All illnesses**	**(** ***n*** ** = 174)**	**(** ***n*** ** = 190)**	**(** ***n*** ** = 163)**	**(** ***n*** ** = 203)**
Home	12.7%	2.6%	7.4%	4.9%
CHW	47.1%	78.9%	50.9%	77.3%
RHC/CMH	40.2%	18.5%	41.7%	17.8%
**Fast breathing**	**(** ***n*** ** = 61)**	**(** ***n*** ** = 66)**	**(** ***n*** ** = 59)**	**(** ***n*** ** = 34)**
Home	6.6%	3.0%	6.8%	8.8%
CHW	50.8%	77.3%	54.2%	55.9%
RHC/CMH	42.6%	19.7%	39.0%	35.3%

CMH, Chikankata Mission Hospital; RHC, rural heath center.

## Discussion

This study has demonstrated the feasibility and effectiveness of using CHWs to provide integrated management of pneumonia and malaria at the community level. Allowing CHWs to dispense amoxicillin to children with nonsevere pneumonia and AL for malaria after the use of RDTs resulted in a significant increase in the proportion of appropriately timed antibiotic treatments for nonsevere pneumonia and in a significant decrease in inappropriate use of antimalarials.

Our study showed a 5-fold increase in the proportion of children with nonsevere pneumonia who received early and appropriate treatment when treated by CHWs in the community instead of the existing system of referral to health centers. This finding adds to the growing evidence of the important role of community-based workers in the management of pneumonia, which has been well documented in South East Asia, as described below, but to a much lesser extent in sub-Saharan Africa. A community-based pneumonia management program in Nepal using female community health volunteers resulted in almost 70% of Nepal's under-five population having access to pneumonia treatment and a reduction of under-five mortality by almost 50% [34]. In Pakistan, case management of acute lower respiratory infections by village level CHWs backed by local health center staff resulted in the reduction of pneumonia-specific and all-cause mortality in children under five [35]. A community-based intervention project in which village heath workers and traditional birth attendants were trained to give mass education about pneumonia and to recognize and treat childhood pneumonia with cotrimoxazole in India also resulted in significant reductions of pneumonia-specific and all-cause mortality [36]. An operational research evaluation project that used nonrandomized design in Senegal showed that CHWs can correctly classify acute respiratory infection and appropriately treat with cotrimoxazole [37].

**Figure 2:**
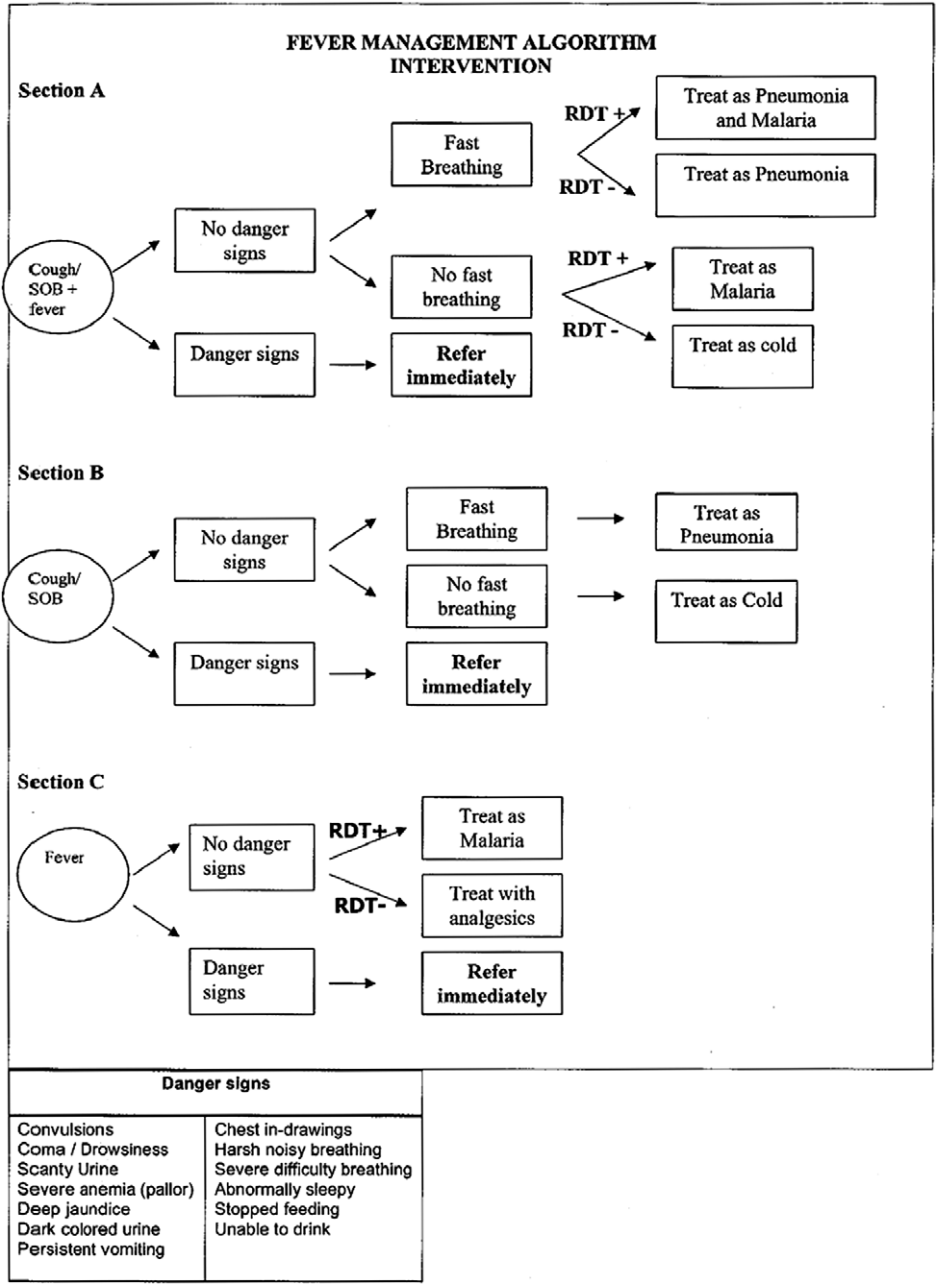
Treatment algorithm for Intervention Community Health Workers.

**Figure 3:**
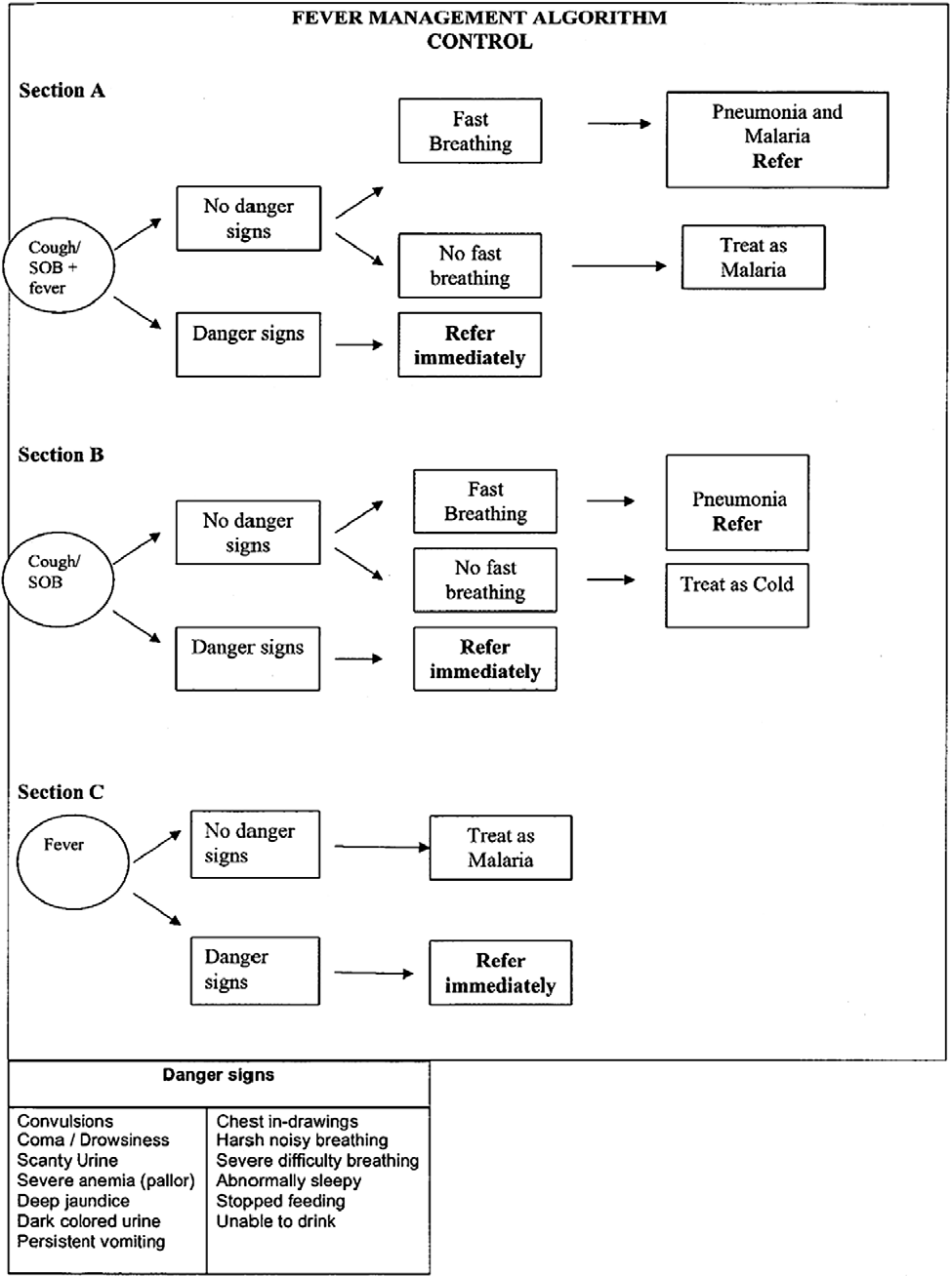
Treatment algorithm for Control Community Health Workers.

We found that adequately trained and appropriately resourced CHWs can perform and interpret RDTs, and give treatment for malaria. This finding is consistent with a recent study in Cambodia [23]. Basing treatment on RDT results led to a 4-fold reduction in the use of AL in this study area; the reduction was as high as 10-fold in the dry months when malaria transmission was quite low. This finding has major implications for malaria treatment since the consequences of malaria overdiagnosis may include poor health outcome due to missed diagnosis of alternative causes of symptoms, exposure to unnecessary medication, wastage of essential medicines, and unnecessary expenditure at both the household and health system levels [38]–[40]. Msellam and colleagues in Zanzibar also found that RDT use was associated with lower prescription rates of antimalarials than symptom-based clinical diagnosis alone [41]. Overdiagnosis of malaria without laboratory support has been widely reported [42]–[45], and the findings of this study support the likelihood that RDT use could substantially reduce the inappropriate use of antimalarials if prescribers adhere to the RDT results. Adherence to the results of the RDTs was very high in this study. This suggests that CHWs are more willing to restrict the use of antimalarials to RDT positive patients [46],[47] than health workers, who frequently opt to treat RDT-negative patients [48]–[50]. However, one study has reported high adherence to RDT results when health workers prescribe AL [51]. In the present study, the refresher training after 6 mo, frequent review and assessment of performance of the CHWs at the RHCs, and a relatively high level of education (68% of the CHWs had secondary education) may have contributed to the high level of adherence to treatment guidelines, which were simple and easy to read and interpret (Figures 2 and 3). Lemma and colleagues in Ethiopia have also shown that the use of AL and RDTs by CHWs is not only feasible but has the potential of reducing malaria transmission and case burden for health facilities [52].

This study is, to our knowledge, the first randomized, controlled trial of the management of both malaria and pneumonia in children at the community level by CHWs using RDTs to differentiate between malaria and pneumonia. In a Care International community-integrated multiple disease management project in Siaya, Kenya, only the performance of CHWs in managing the multiple diseases was evaluated [25]. Degefie and colleagues have just reported the findings of an evaluation of a project using volunteers to provide treatment for childhood diarrhea, malaria, and pneumonia in a remote district in Ethiopia. The volunteers in this project did not use RDTs and the investigators used a pre–post study design [24].

With the use of RDTs in this study, less than 30% of children with a clinical diagnosis of nonsevere pneumonia were confirmed to also be infected with malaria, compared to almost 90% of the children in the control arm, in which malaria and nonsevere pneumonia diagnoses were syndromic. Without the use of RDTs, most children diagnosed with pneumonia will also be classified as having malaria and will receive antimalarial drugs [19]. Ansah and colleagues in Ghana showed that using RDTs led to a significant reduction in overprescription of antimalarials and better targeting of antibiotics [53]. Providing effective and safe oral treatment for the community-based treatment of malaria will substantially improve access to care for children in malaria-endemic areas and will undoubtedly save lives. However, because of the overlap in clinical presentation for malaria and pneumonia, providing CHWs with malaria-specific treatment (AL) but no effective antibiotics for treating pneumonia or a means to distinguish the two, will undoubtedly lead to pneumonia treatment delays. Failure to comply with referral because caregivers did not think that the child was very sick or has been presumptively treated for malaria was seen in the present study; this has been documented elsewhere [54],[55]. Providing CHWs with the means to treat malaria but not pneumonia increases the risk of treatment delay and progression to more severe disease for children with pneumonia.

Our study has a number of strengths including the cluster randomized design, large sample size, accounting for clustering in analysis, additional training program and supervision of CHWs, use of a simple algorithm for diagnosis of the two diseases, and a 12-mo duration, which allowed for seasonal variation of childhood illnesses.

A major limitation of this study was an imbalance in the number of individuals enrolled between the study arms. Since the CHW and child characteristics (including time to health care seeking) were similar in both arms, there is evidence that randomization was not compromised. Clusters were only matched in pairs according to the distance between the community health post and the health center. At the time the study was designed, there were no data available on the size of the catchment areas of the different community health posts. Since utilization of services and health-seeking practices are multifaceted and influenced by many factors [56], it is likely that cluster randomization could not address all of these factors, and hence the resulting imbalance. The fact that more CHWs in the control arm considered themselves as full-time workers and therefore available to see patients most of the day may also have contributed to the larger number of patients seen in the control arm. In favor of this interpretation was the finding that unavailability of CHWs was the most common reason for caregivers not using the services of CHWs. There were twice as many children classified with pneumonia in the intervention arm relative to the control arm. As confirmed by the presenting complaints and postintervention household surveys, caregivers who suspected that their children had pneumonia (due to complaints of fast/difficult breathing) preferentially brought them to the intervention CHWs because they knew that amoxicillin was available. Caregivers in the control arm who suspected that their children had pneumonia bypassed the CHW and went straight to the rural health center. The population of the intervention and control arms was found to be similar; thus the imbalance in the number and cases seen is most likely due to health-seeking practices in response to the intervention and potentially unequal distribution of the catchment population sizes between the two study arms. There was no indication of any significant “contamination” of control caregivers seeking care from intervention CHWs.

Improving access to care for remote communities through the implementation of community case management of disease is an important new focus for global health policy. Community case management of pneumonia is an effective approach to reducing child deaths in countries faced with insufficient human resources for health [34],[57] and a feasible, effective strategy to complement facility-based management for areas that lack access to facilities [58]. In addition to optimizing the management of malaria and pneumonia, community case management should also integrate treatment of dehydration due to diarrheal disease with oral rehydration therapy, as was the practice in our study site in rural Zambia, and should also integrate the use of zinc. Future efforts should focus on the incorporation of life-saving interventions for severe disease at the community level including rectal artesunate for severe malaria [59] and amoxicillin for severe pneumonia [60].

With improved point-of-service technologies such as RDTs for malaria, the skills of CHWs can be substantially enhanced. The use of RDTs by CHWs is likely to receive the approval of community members since providers with diagnostic capacity are generally preferred [61]. This study adds to the growing evidence that integrating community case management of pneumonia and malaria is feasible, opening the door to evaluations of the treatment by CHWs of other major diseases of children. Much can be done at the community level to save the lives of children in sub-Saharan Africa [62].

## Supporting Information

Text S1 Protocol.
(0.33 MB DOC)

Text S2 CONSORT checklist.
(0.19 MB DOC)

Text S3 CHW training manual.
(0.31 MB DOC)

Text S4 CHW RDT training manual.
(1.93 MB DOC)

## Abbreviations

AL: artemether-lumefantrine
ACT: artemisinin-based combination therapy
CHW: community health worker
CI: confidence interval
RDT: rapid diagnostic test
RR: risk ratio
