# Supplementary material for: Community Case Management of Fever Due to Malaria and Pneumonia in Children Under Five in Zambia: A Cluster Randomized Controlled Trial
Source: PLoS Med. 2010 Sep 21;7(9):e1000340. doi: 10.1371/journal.pmed.1000340 (PMC2943441; doi:10.1371/journal.pmed.1000340)
Supplement: Text S1 — Protocol. (0.33 MB DOC) [file pmed.1000340.s001.doc]

***Zambia integrated management of malaria and pneumonia STUDY (ZIMMAPS)***

**STUDY PROOCOL**

**A Collaborative Community Based Study Between:**

**Center for International Health and Development, Boston University School of Public Health**

**Mazabuka and Siavonga District Health Management Teams**

**Ministry of Health**

**Chikankata health services**

# ABbreviations

| **ACTs** | **Artemisinin-based combination therapies** |
| --- | --- |
| **AIDS** | **Acquired Immunodeficiency Syndrome** |
| **ALRI** | **Acute Lower Respiratory tract Infections** |
| **BUSPH** | **Boston University School of Public Health** |
| **CER** | **Cost-effectiveness Ratio** |
| **CHS** | **Chikankata Health services** |
| **CHW** | **Community Health Worker** |
| **CIHD** | **Center for International Health and Development** |
| **CPTs** | **Care and Prevention Teams** |
| **CRFs** | **Case Report Forms** |
| **DC** | **Data Collector** |
| **DHMT** | **District Health Management Team** |
| **DS** | **Data Supervisor** |
| **DSMB** | **Data Safety and Monitoring Board** |
| **GEE** | **Generalizing Estimating Equation** |
| **HCC** | **Health Center Committee** |
| **HIV** | **Human Immunodeficiency Virus** |
| **ICC** | **Intra-cluster Correlation** |
| **IMCI** | **Integrated Management of Childhood Illness** |
| **MOH** | **Ministry of Health** |
| **NGO** | **Non-governmental Organization** |
| **NHC** | **Neighborhood Health Committee** |
| **NMCC** | **National Malaria Control Center** |
| **OPD** | **Outpatient Department** |
| **PHC** | **Primary Health Care** |
| **RDTs** | **Rapid diagnostic tests** |
| **RHC** | **Rural Health Center** |
| **ZIMMAPS** | **Zambia Integrated Management of Malaria and Pneumonia Study** |

# 1. Study Synopsis

# The Zambia Integrated Management of Malaria and Pneumonia Study (ZIMMAPS) will be a cluster randomized controlled trial to demonstrate the effectiveness and feasibility of community-based management of pneumonia and malaria by community health workers (CHWs) in a rural district of Zambia. The CHWs will be aided by the use of rapid diagnostic tests (RDTs) to differentiate between fever due to malaria and fever due to other causes, especially pneumonia, with the overarching goal of enhancing the capacity of CHWs to provide early, effective and appropriate treatment to children under five in areas of particularly poor access to facility based health care.

The study will be conducted in the Chikankata Health Services (CHS) area, which covers parts of Mazabuka and Siavonga Districts of the Southern Province of Zambia. It will be a collaborative venture between the Center for International Health and Development (CIHD) of Boston University School of Public Health (BUSPH), Mazabuka and Siavonga District Health Managements (DHMTs), the Ministry of Health (MOH) and CHS, a non-governmental organization (NGO).

Existing supported CHWs in the study area will be trained in the assessment and classification of children between six months and five years of age presenting with fever and/or cough/difficult breathing. In the intervention arm, CHWs will be supplied with RDTs, Coartem® and amoxicillin. The intervention CHWs will be trained to use RDTs in patients with reported fever and provide those with a positive result with Coartem®; and patients suspected of pneumonia (based on fast breathing) will be treated with amoxicillin as per the standard of care at health facilities and monitored. In the control group, no RDT will be performed. The CHWs will be supplied with Coartem to treat malaria/febrile illness as per the integrated management of childhood illnesses (IMCI) guidelines and patients suspected of pneumonia will be referred to the health facility for treatment as per the current practice. Data collectors will routinely visit CHWs to collect data on their consultations and follow-up patients treated by CHWs in their homes..

# 2. BACKGROUND AND RATIONALE

Pneumonia and malaria are the two leading causes of morbidity and mortality among children under five in sub-Saharan Africa. Pneumonia accounts for nearly one-fifth of childhood deaths worldwide, with approximately 2 million children under five dying each year. The majority of deaths occur in Africa and Southeast Asia.^1^ Childhood malaria, and in particular *P. falciparum* malaria, remains a major public health problem, with an estimated one million children dying each year of malaria and its complications.^2,3^ Recent estimates of the global distribution of clinical episodes of *P. falciparum* malaria suggest that the burden of malaria in Africa may even be grossly underestimated.^4^

Malaria is the most significant health problem in Zambia accounting for, by far, the greatest number of pediatric outpatient consultations and hospital admissions. The National Malaria Control Center (NMCC) estimates that malaria is responsible for nearly 4.3 million clinical cases and an estimated 50,000 deaths per year^5^.

# A large proportion of these deaths could be prevented through early, appropriate and low-cost treatment of sick children. The IMCI algorithm represents a notable attempt to extend these basic health care services to sick children. IMCI was designed to be implemented in primary health care clinics by health workers who only have a rudimentary level of education or medical training.

Improvements in care at health facilities through IMCI and other initiatives are necessary, but have been found not to be sufficient^6^. Children from the poorest families are significantly less likely to be brought to health facilities, and may receive lower quality care once they arrive^7,8^. Even where impressive gains are made in the quality of care in health facilities, the level of care seeking from these same facilities remains suboptimal ^9,10^ and these services are geographically not accessible to the majority of the children who need them. The health needs of large numbers of sick children in developing countries are provided through the informal sector (which may include CHWs and drug sellers) or traditional healers^11^, rather than through primary health care facilities where IMCI protocols might be in use.

Since access to health services is limited in Sub-Saharan Africa and a majority of African children die at home^12^, new and innovative approaches to reducing childhood mortality will require interventions implemented at the community level.

Consequently, child survival programs have placed increasing importance on early and appropriate treatment of malaria and pneumonia within the community, and preferably within the child’s own home. Two recent studies from Bangladesh and Nepal demonstrated the feasibility and effectiveness of training CHWs to manage acute lower respiratory tract infections (ALRI)^13,14^. In both cases, this model of care led to significant reductions in childhood mortality, and, importantly, was well accepted by community members. A meta-analysis of studies from eight Asian and one African nation (Tanzania) found that community-based management of pneumonia reduced mortality by 36% among infants and children.^15^ Similarly, a number of studies have shown that CHWs can be trained to provide effective malaria case management in the community^16,17^. In a large community-based, cluster randomized trial in northern Ethiopia, CHWs were trained to identify malaria on clinical grounds and to administer chloroquine in the field. This approach generated an impressive 40% reduction in all-cause mortality, with the greatest reduction due to reduced malaria deaths.^15^

# At present most malaria illnesses are diagnosed on highly non-specific clinical grounds alone, without the benefit of diagnostic tools. Consequently, antimalarial drugs are administered presumptively to children with history of fever. This practice leads to substantial over treatment and may accelerate the evolution of anti malarial drug resistance, as well as wasting scarce resources and delaying diagnosis and treatment of other febrile illnesses especially pneumonia. With Zambia’s recent decision to replace sulfadoxine-pyrimethamine (Fansidar®) with artemether-lumefantrine (Coartem®) for use by CHWs, the potentially harmful effects of presumptive use of this drug might become even more pronounced.

In an attempt to provide an effective alternative to the traditional method of malaria testing, malaria RDTs have been developed. While RDTs can be effective in clinical settings when used by trained personnel, they also have great potential in rural areas with limited access to health facilities and health professionals^18^. RDT use by CHWs at the village level presents a tremendous opportunity to improve malaria diagnosis and treatment. It should lead to making a definitive diagnosis in a much greater proportion of cases and significant cost savings by avoiding unnecessary use of expensive artemisinin-based combination therapies (ACTs) and thus may help reduce the development of drug resistance. Given the high cost of ACTs, the Zambia MOH has adopted strategies to improve malaria diagnostics (microscopy and the use of RDTs to rationalize the use of ACTs in peripheral clinics. The Quality Assurance Project in partnership with the Special Programme for Research and Training in Tropical Diseases, the World Health Organization Regional Offices for the Western Pacific and Africa (AFRO) and the Zambian MOH has developed and tested an RDT job aid to enable CHWs safely and effectively use RDTs. The Zambian MOH is currently discussing the mechanism of how to deploy RDTs to improve malaria diagnosis at the community level.

# A number of global health organizations, including the World Health Organization, have strongly advocated the use of CHWs to deliver basic health care in the community and to facilitate referral to primary health facilities. The symptoms of malaria and pneumonia overlap to a great extent, and therefore to adequately treat the sick child, these community strategies need to address the malaria-pneumonia symptom overlap and manage both conditions ^19^. However, there are limited studies that have evaluated CHWs managing both ALRI and malaria; and no study has thus far carefully evaluated CHWs delivering artemisinin-based combination therapy. The potential benefit of RDT in improving malaria diagnosis before treating with ACTs by CHWs also needs to be proven.

The purpose of this project is to demonstrate the effectiveness and feasibility of training CHWs and supplying them with RDTs, Coartem® and amoxicillin to treat both pneumonia and malaria at the community level, with the overarching goal of enhancing the capacity of CHWs to provide early, effective and appropriate treatment to children under five in areas of particularly poor access to facility based health care. We decided to work with CHWs because the Zambia MOH recognizes the role of CHWs in providing cost effective and quality health care as close to the family as possible and is committed to supporting and strengthening the implementation of community based health care. The MOH with support from other partners has developed a reference manual for CHWs. This manual is being used by DHMTs, which in partnership with NGOs are running CHW programs in many parts of the country. The project will be conducted in close partnership between CIHD, the Zambian Ministry of Health and the Salvation Army World Service Office, a NGO working in Southern Zambia. We believe that the dual disease management model will prove feasible and effective, and offer a new model of care that will be highly relevant in Zambia and throughout sub-Saharan Africa.

We do recognize the challenges inherent in wide-scale use of CHW programs, which may include maintaining and ensuring high level of performance, development of less complex and culturally appropriate job aids^20^, retention, motivation and incentives, health systems support (in the area of provision of drugs and supplies, supervision and referrals) and community support. These issues will be addressed in designing the study. We hope that the study will provide useful lessons for scaling up this approach.

# 3. OBJECTIVES AND OUTCOME VARIABLES

**3.1 Main Objective**

- To demonstrate the effectiveness and feasibility of integrated management of fever (malaria and pneumonia) at the community level using CHWs with the aid of RDTs.

**3.2.1 Primary Objectives**

- To determine whether the integrated management of malaria and pneumonia by CHWs at the community level increases the proportion of children under five years old who receive early and appropriate treatment for pneumonia as compared to children referred to health facility.
- To determine the extent, to which the use of RDTs by CHWs reduces the use of Coartem® in the management of children with acute fever, compared with management based on clinical criteria only.

**3.2.2 Primary Hypotheses**

- The proportion of children with fast breathing who receive early and appropriate treatment for pneumonia will be increased by at least 20% among CHWs trained to treat both malaria and pneumonia.
- The proportion of children presenting with fever who receive Coartem® will be reduced by at least 20% among CHWs trained in the use of RDTs.

#### 3.3.1 Secondary Objectives

- To measure the proportion of children managed in each study arm who do not respond to treatment (treatment failures).
- To measure the cost-effectiveness of having CHWs manage malaria using Coartem® with RDTs compared to without the use of RDTs.
- To measure the proportion of acute febrile illnesses in children which are RDT-positive
- To determine the burden of non-malaria febrile illness among a rural population of children reporting fever

**3.3.2 Secondary Hypotheses**

- The use of RDTs in the management of febrile illness coupled with pneumonia treatment at the community level instead of referring will reduce the rate of treatment failure.
- The use of Coartem® by CHWs will be more cost effective when guided by RDTs than when guided by clinical criteria alone.
- The use of RDTs will demonstrate that a significant proportion of children presenting with acute febrile illness and who would otherwise be treated for malaria do not in fact have malaria.

**3.4 Primary Outcomes**

- Proportion of children with pneumonia who receive early (start treatment with amoxicillin within at least 24 hours and at most 48 hours of the onset of illness) and appropriate (correct dose for age for 5 days) treatment.
- Proportions of febrile children in each arm who are treated with Coartem®

**3.5 Secondary Outcomes**

- Proportion of children treated who do not respond to treatment (experience treatment failure – defined here as any of the following:
  - Failure to clear symptoms by day 5 after treatment.
  - Subsequent referral to health center or hospital despite treatment.
  - Development of severe signs despite treatment
  - Use of additional antibiotics or antimalarials beyond those prescribed and dispensed by the CHW.
  - Serious adverse event considered possibly or probably related to drugs.
- Costs of treatment and cost associated with the use of RDTs by CHWs
  - Initial and ongoing training costs per CHW
  - Costs of RDT kits/reagents
  - Costs per course of Coartem®
  - Other costs associated with the use of RDTs by CHWs
  - Cost associated with referral
- Proportion of febrile children with a positive RDT result

**3.6 Other Outcome Variables**

- Proportion of negative RDT cases which receive ACTs from CHW or another malarial treatment from another source.
- Proportion of negative RDT cases with fast breathing and treated for pneumonia.
- Proportion of children with fast breathing whose symptoms and signs resolve without antibiotics.
- Proportion of children with pneumonia referred who comply with referral within 24 hours
- Proportion of children who experience adverse events attributable to the use of RDTs, such as severe bruises, skin infections, and prolonged bleeding following finger pricks
- Proportion of sick children correctly classified by CHW
- Proportion of sick children prescribed appropriate treatment for malaria/pneumonia according to the treatment algorithm
- Proportion of children treated for malaria/pneumonia who experience adverse events
- Proportion of drug and/or RDTs supply properly accounted for (consistency between stock in hand from stock summary forms and stock consumed based on consultation/treatment forms)
- Proportion of caretakers who take children to a CHW or health facility within 24/48 hours of onset of fever and or cough/difficult breathing.

.

**4.0 METHODS**

# 4.1 Study Site

The study will be conducted in the Chikankata Health Services area covering parts of Siavonga and Mazabuka Districts, which are located in the Southern Province of Zambia. The estimated population of the area is 70,000. The people of Siavonga and Mazabuka are Tonga.

The area is a typical rural area with poor road networks. Some parts of the area are inaccessible particularly during the rainy season as streams cut off some villages for at least two months of the year. Only two towns in the area are connected to the national power grid. The area has one hospital, the Chikankata Hospital with a hospital affiliated health center and five rural health centers (RHCs), two in Siavonga district and three in Mazabuka district. The hospital is owned by the Salvation Army Church and the RHCs by the government. Some of the RHCs do not have the full complement of staff (clinical officer, environmental health technician, nurse, and midwife). The rural health facilities are on average 30 kilometers away from Chikankata Hospital. Most services at the hospitals and RHCs are free, including all services for under-fives and pregnant women.

Data from the DHMTs show that the leading causes of death for children under five in the area are malaria, malnutrition, pneumonia, and diarrhea^21,22^ Malaria has consistently been the number one cause of consultation at all levels of health care in the area. The incidence of malaria in under fives, over the last few years ranges from 900 to 1200 per 1000 of the under five population. Similarly, the incidence of pneumonia and respiratory tract infections (non-pneumonia) is 70 to 80 per 1000 and 500 to 600 per 1000 respectively.

CHS is actively running a Primary Health Care (PHC) program which has a mobile and outreach services unit. The Mobile and Outreach Services unit support and supervise the RHCs and a number of community based workers, which include traditional birth attendants, community based distributors who distribute family planning devices, community nutrition demonstrators and the CHWs. The community-based workers normally work together and operate from a community health post structure built by the community.

There are 49 trained CHWs manning 43 community health posts in the area and supported by CHS. The initial CHW training lasted for twelve weeks, which included six weeks of classroom work and six weeks of practical. The responsibilities of the CHW involve treating minor illness including uncomplicated malaria, disease prevention, health education and community sensitization and mobilization, especially for outreach services. They work within the catchment area of health facility. They are supervised and supported by the mobile and outreach team of the PHC and staff of the RHCs. Most of the CHWs collect their community health worker kits monthly from Chikankata Hospital but a few collect from the supervising RHC. They submit monthly returns to Chikankata and the RHC on a specially designed form “CHW: Village Health Center Monthly Aggregation”. This form collects aggregated information on diseases seen, referrals to rural health centers and hospital, births, deaths, drug and material supply and consumption. There is no diagnosis of pneumonia listed on the form.

There is no formalized incentive package for the CHWs but CHS provides some incentives from time to time when resources are available. Such incentives include bicycles, umbrella and stationery. They also receive some incentives from the communities. These incentives are mostly in-kind and include items such as eggs, maize, and chickens. The community also assists them by organizing help for them on their farms and household tasks. Attrition rate of the CHWs is very low. The Chikankata Community Health Program follows the guidelines provided in the Zambia MOH reference manual for CHW “Community Health Workers Handbook” in running its program.

There are also neighborhood health committees (NHCs). These are groups of community-selected members who help manage community health services at the community level and hold community meetings to link people and health care providers. The chairpersons of these NHCs make up health centre committee (HCCs) that help in planning health activities and support the management of RHCs. In addition to these community groups, there are care and prevention teams (CPTs) formed in the wake of the Human Immunodeficiency Virus (HIV) /Acquired Immunodeficiency Syndrome (AIDS) epidemic who assist people infected and affected by HIV/AIDS. They also support referral, nutrition supplementation, care and support of the sick.

# 4.2 Study design

The study will be a cluster-randomized controlled trial. A study cluster will be defined as the catchment area of a community health post. CHWs work from a community health post with well defined catchment area and population within a health facility catchment area and are supervised by the staff of the health facility. The clusters (community health post catchment area) will be matched in pairs according to the health facility catchment area they belong to, the distance from the health facility and the population size. Randomization will then be done from among the matched clusters to select the intervention and the control arms. CHWs in both the intervention and control arms will receive training in the assessment and classification of children presenting with fever and/or cough/difficult breathing. The CHWs in the intervention arm will be supplied with RDTs, Coartem® and amoxicillin. The intervention CHWs will be trained to use RDTs in patients with reported fever and provide those with a positive result with Coartem® while patients suspected of pneumonia (based on fast breathing) will be treated with amoxicillin as per the standard of care at health facilities and monitored. Those with negative RDT result will be treated with antipyretic and asked to return if they do not get better after 48 hours or condition deteriorates. In the control group, no RDT will be performed. The CHWs will be supplied with Coartem to treat malaria/febrile illness as per the IMCI guidelines and patients suspected of pneumonia will be referred to the health facility for treatment as per the current practice. The supply of the drugs and RDTs will come through the normal MOH sources. The study will utilize specialized field workers (data collectors and data supervisors) to visit CHWs to collect data on their consultations and follow-up children treated by CHWs. Consent will be sought from caregivers to allow us collect data by which to assess the effectiveness of the intervention. Caregivers in both the intervention and control arms are not obliged to receive care from the CHWs. They are free to go to any of the health facilities in the study area instead of a CHW. The study will collaborate with the MOH and the DHMTs to ensure that the health facilities in the study area have RDTs, Coartem and amoxicillin.

**4.3 Sample Size**

This study will be a cluster randomized trial at the level of the community health post. As a result, we need to take the intracluster correlation within individuals receiving treatment at each community health post in both the calculation of sample size and data analysis.

The two primary study hypotheses were stated earlier: one for the treatment of children with fast breathing and one for the treatment of children with fever. We will calculate a sample size for each of the study hypothesis

There are 43 community health posts in the study area. This will be a two-arm study so we will have 21 community health posts available for each arm with a 1:1 allocation. All of the calculations are made using the assumption of 80% power to show differences and a two side alpha of 0.05.

The sample size for a cluster randomized trial is based upon the intracluster coefficient (ICC). As we do not have accurate information on the expected ICC for the outcomes in this study, the sample size is presented as a series of sample size calculations given a range of the ICC values which we would expect to see.

## Treatment of children with fast breathing

We hypothesize that the proportion of children with fast breathing who receive early and appropriate treatment for pneumonia will be increased by at least 20% among CHWs trained to treat both malaria and pneumonia. Currently it is estimated that only 50% of children receive early and appropriate treatment for pneumonia. Therefore, we would be looking at increasing this to 70%.

The table below shows the total clusters, sample per clusters and total sample size for each level of ICC.

**Sample size necessary to detect a 20% increase in the proportion of children receiving early and appropriate treatment for fast breathing for various ICCs**

| Intracluster coefficient | Total Clusters | Sample per cluster | Total sample size |
| --- | --- | --- | --- |
| 0.05 | 36 | 6 | 216 |
| 0.07 | 36 | 6 | 216 |
| 0.09 | 38 | 6 | 228 |
| 0.11 | 38 | 6 | 228 |
| 0.13 | 36 | 7 | 252 |
| 0.15 | 38 | 7 | 266 |
| 0.17 | 36 | 8 | 288 |
| 0.19 | 38 | 8 | 304 |
| 0.21 | 38 | 9 | 342 |
| 0.23 | 38 | 10 | 380 |
| 0.25 | 38 | 12 | 456 |

At the largest ICC of 0.25, 38 clusters enrolling 12 subjects each, a total of 456 subjects would be necessary to show a difference between the intervention and control arms. At the smallest ICC of 0.05, the total sample size would be only 216 subjects who would be recruited from 36 clusters with 6 subjects per cluster. Therefore, within this expected range of ICC, we would need to enroll between 216 and 456 subjects. Since the ICC cannot be precisely estimated we would enroll the 456 subjects. We anticipate a drop out rate of 15%; hence we will increase our sample size to 524 children with fast breathing.

**Treatment of children presenting with fever**

Currently 100% of children seen with fever should receive Coartem treatment. We hypothesize that with the use of an RDT, the proportion of children receiving Coartem treatment would be reduced to 80%.

**Sample size necessary to detect a 20% decrease of children not receiving Coartem for various ICCs**.

| Intracluster coefficient | Total Clusters | Sample per cluster | Total sample size |
| --- | --- | --- | --- |
| 0.05 | 38 | 2 | 76 |
| 0.07 | 40 | 2 | 80 |
| 0.09 | 42 | 2 | 84 |
| 0.11 | 34 | 3 | 102 |
| 0.13 | 36 | 3 | 108 |
| 0.15 | 36 | 4 | 144 |
| 0.17 | 36 | 5 | 180 |
| 0.19 | 38 | 6 | 228 |
| 0.21 | 38 | 12 | 456 |
| 0.23 | 40 | 33 | 1320 |
| 0.25 | 46 | 36 | 1656 |

At the largest ICC of 0.25, 46 clusters enrolling 36 subjects each for a total of 1656 subjects would be necessary to show a difference between the intervention and control arms. Since we do not have 46 clusters, and no feasible increase in subjects per arm will allow us to reduce the total clusters to 42, we would be underpowered with an ICC of 0.25. However, we would be able to meet the study enrollment needs at every other ICC. At the smallest ICC of 0.05, the total sample size would be only 76 subjects being recruited from 38 clusters with 2 subjects per cluster. Therefore, within these likely ranges of ICC we would need to enroll between 76 and 1320 subjects. Following the logic above we would enroll 1320 subjects. We anticipate a drop out rate of 15%; hence we will increase our sample size to 1518 children with fever.

All of the 49 CHWs supported by CHS will be used.

20 households per cluster will be selected through multi-stage sampling. 420 caregivers will therefore be interviewed. Since the household survey will be repeated at the end of the intervention, a total of 840 caregivers will be interviewed.

The total sample size for the study will be 2931 (524 + 1518 + 840 + 49)

### Study Population and Recruitment

### Children between six months and five years who present to a CHW at the community health post with fever and/or cough/difficult breathing will be assessed, classified and managed. Six months was selected as the cut off because Coartem® is not recommended for younger children weighing less than 5 kg and also because of the difficulty of assessing very young children for pneumonia. Children outside this age range and adults will be managed according the current treatment guidelines for CHWs and will not be followed up by study personnel. Any child with severe illness or signs of severe malaria or pneumonia will be referred as per standard of care and excluded from enrollment into this study.

###### Inclusion criteria:

Children with the following characteristics will be considered for study inclusion:

- Age between 6 months and 5 years
- Present to CHW with history (reported) of fever.

### Present to CHW with cough or difficult breathing

### *Exclusion criteria:*

Children with any one or more of the following conditions will be excluded:

- Age below 6 months or above 5 years
- Presence of signs and symptoms of severe illness
  - Convulsions
  - abnormally sleepy or coma
  - persistent vomiting
  - unable to drink or feed
  - chest in-drawings
  - severe pallor
- Known sensitivity to Coartem® and/or amoxicillin

The CHWs will keep register of all patients seen as part of standard care. From this register, the CHWs will extract information to complete identification and baseline form for every child with fever or/and fasting breathing. This form will be used by data collectors to locate patients for the follow-up visits and also to measure the accuracy of classification and appropriateness of treatment.

In the intervention arm, children 6 months to 5 years whose caregivers refuse RDT testing will not be followed up. However, we will keep track of them and measure the proportion of RDT refusals.

The option for a caregiver to go to a health facility to receive care in place of being cared for by the CHW is an accepted norm in the communities. However, existing community based health educators in the area (NHCs, child survival facilitators, community based distributors, traditional birth attendants, growth monitors) in their interaction with community members will emphasize that they have this option.

### 4.5. Roles and responsibilities of community health workers

The primary role of the CHWs is to serve as the households’ first point of contact with the health system. They will classify the child’s illness and provide specific drug treatment.

The key responsibilities will include:

- taking a history from the caretaker
- assessing the child including respiratory rate, temperature and the presence of danger signs;
- treating or referring as appropriate
- dispensing specific medication and observing the child taking the first dose
- providing the caretaker with instructions for the remainder of the treatment course, as well as for care of the sick child in the home.
- counseling caregivers to return if condition deteriorates or experience any adverse effects
- conveying a sense of urgency to the caretaker when a referral is indicated, administering start dose of medication if indicated, and providing a referral slip.
- managing a stock of medications and supplies
- maintaining simple records and registers
- conducting community education

In the intervention arm, the CHWs will also perform and interpret RDTs

#### 4.6 Minimum requirements for CHWs

CHWs involved in this project must be literate, numerate, and able to write and manage simple calculations. After training, they must be competent in assessing respiratory rate and recognizing danger signs, and following the management classification charts. They must be acceptable to caregivers as well as to communities. CHWs must be available and willing to see patients outside regular hours in cases of emergency. Finally, they must be honest and willing to follow the protocol.

**4.7 Training of community health workers**

The CHWs will be trained to classify and treat children with pneumonia or malaria or both and also manage stock of appropriate drugs and supplies. The CHWs in the intervention areas will also be trained to perform RDTs. They will also require skills to be able to accurately complete the relevant registers and data collection forms as well as counseling of caregivers and general health education. The training will last for one week and a training manual will be developed for this purpose. The training manual will be adapted from Zambia’s “Community Health Workers’ Handbook: A Reference Manual for the Community Health Workers”. The training will be conducted by a training team experienced in IMCI skills training and will be drawn from both Mazabuka and Siavonga districts with support from the Provincial CHW training team. The training will be conducted in groups of 15-20 at Chikankata hospital.

The training course will cover the following elements:

- taking basic clinical history
- basic examination skills with emphasis on counting respiration, assessing lower chest in-drawings and taking temperature
- recognition of general danger signs, classification of sick child according to the classification system for malaria and pneumonia
- treatment – including side effects of the drugs
- performing and interpreting RDTs.
- referral procedures
- provision of instructions and counseling to caregivers (including home care, referral, importance of completion of course of drugs, need to give Coartem**®** with fatty meal )
- drug stock management
- record keeping

The training will use a variety of methodologies including lectures, presentations, discussions, case studies, demonstrations, practice, role-plays, and video shows. The CHWs will take quality time to go through and practice the use of a diagnostic tool that will be developed to assist them to classify cases. The trainer/supervisors will follow up the CHWs one month after the initial training to ensure that they acquired the necessary skills. There will be refresher courses every six months and will last 2 days.

There will be a half day session for the intervention CHWs on hands-on introduction to RDT use. This will cover performing and interpreting the test – cleaning the finger with swab, pricking with a lancet, drawing blood with the pipette, squeezing the blood from the pipette into the well, adding the buffer and reading the results. The training will also cover the need to maintain aseptic techniques and to protect themselves from being pricked with the lancet; and disposal of waste products including the lancet, pipette, and device. They will also be taken through procedures of what to do if they accidentally prick themselves including immediate washing of wound with antiseptic soap and plenty of water.

CHWs in the Philippines and Laos have been trained to perform and interpret RDTs using job aids with great success^23^.

#### 4.8 Training of health center personnel in IMCI

In order to support and supervise the CHWs during the implementation of the study, rural health center personnel in the study area will undergo full IMCI training. The study will support this training because the ongoing IMCI training for all health personnel by the MOH in the country has been lagging behind in the Southern Province where the study area is located. This will also enable them to deal effectively with cases that will be referred from the CHWs. The training will cover classification and management of IMCI diseases, supervisory and monitoring skills including monitoring of drug use by the CHWs. The training will also cover their role and responsibilities in the data capture system that will be developed to capture data at the health facilities to measure some of the study outcomes. The Provincial IMCI Training Team and the study coordinator will conduct the training.

**4.9 Training of Data collectors and Data Supervisors**

This study will use 12 data collectors (DCs) (2 per cluster) and 2 data supervisors (DS). Each DS will be responsible for 6 DCs. The DCs and the DS will be trained to be able to conduct the follow-up visits and collect data using the case report forms that will be developed. They will be trained to take basic clinical history and perform basic examination skills with emphasis on counting respiration, assessing lower chest in-drawings and taking temperature. The training will also cover the protection of human subjects and confidentiality. The training will provide the data team with the necessary communication and interviewing skills so that they can conduct successful interviews and collect useful data. During the training, the participants will be taken through the case report forms question by question, explaining each thoroughly and detailing the information required. Training methods that will be used will include, discussions, practice and role plays. The case report forms will be translated into the local language and piloted during the final days of the training. The training will last five days. The training will be conducted by a team, made up of Dr Yeboah-Antwi (PI) and the two Zambian Co-investigators.

Four others will be trained as DCs but will be put on “shelf” and only be used to replace any of the twelve whose performance falls below expectations or who resigns during the course of the project. All the trainees will be given a training allowance.

#### 4.10 Development of appropriate treatment algorithm and job aids

Appropriate treatment algorithm and job aids will be developed to aide the CHWs to classify and manage pneumonia and malaria. These tools will be adapted from existing tools and piloted before their use. The tools will walk the CHW systematically through what he/she should ask or look for and the action that needs to be taken. The job aids will include a chart of danger signs (very sick child) and how to perform and interpret RDTs based on the manufacturer’s instructions. The training of the CHWs will emphasize the use of these tools.

#### 4.11 Drug Supply and Management

Currently, CHWs receive community health worker kits prepared by the Central Medical Stores in Lusaka through the districts and the rural health centers. In the Chikankata Health Services area, CHWs in RHC catchment areas of Mazabuka collect their kits from Chikankata Hospital after the latter has collected them from the Mazabuka DHMT. In few cases, the RHC staff collects the kits from Chikankata Hospital and distributes them to the CHWs. For CHWs in RHC catchment areas of Siavonga, they get their supply from Siavonga DHMT through the RHCs. But sometimes they collect the kits from the Chikankata Hospital.

For the purpose of this study, both the Mazabuka and Siavonga DHMTS have agreed to streamline the supply system. Both DHMTs will allow CHS to distribute the kits to the CHWs. This means that CHS will collect the kits from the respective DHMTs and stock them at the Chikankata Hospital from where it will be distributed to the CHWs. The Coartem**®,** amoxicillin and the RDTs will be packaged as a supplementary pack at Chikankata Hospital under the supervision of the hospital pharmacist and distributed to the CHWs with the standard community health worker kit on a monthly basis. The Coartem**®,** amoxicillin and RDTs will come from the usual MOH source for supplying health centers.

Inventory forms will be designed to monitor the supply of medicines and RDTs. Even though CHWs will be supplied on a monthly basis, restocking of essential medications and supplies will occur from Chikankata hospital if the CHW runs out. The RHCs staff responsible for supervision will hold a 1 – 2 month buffer stock of the drugs and RDTs and will restock a CHW’s supply if the need arises during supervision. This system is intended to avoid stock-outs. There will be special section during the training of CHWs on drugs and RDTs management and storage as well as dosing of drugs.

The Study team shall collaborate with the MOH and DHMTs to ensure that the health facilities in the study area have Coartem**®,** amoxicillin and RDTs in stock.

**4.11.1 Rationale for choosing Amoxicillin**

The decision to use amoxicillin was made in conjunction with the IMCI unit of the Zambian MOH. We chose amoxicillin based on the comparative therapeutic advantages and disadvantages of several candidate antibiotics, local patterns of use, and restrictions on the categories of antibiotics that can be used in community settings according to current Zambian health policies. It is one of the drugs recommended by the Zambia National treatment guidelines for pneumonia. Amoxicillin has a long safety record, is readily available in Zambia, is inexpensive, widely accepted by patients and clinicians, and has excellent bioavailability when taken orally.

The very few serious side effects to amoxicillin that occur are due to hypersensitivity. Since it will be given in an oral form, this form of reaction is not anticipated. Less acute reactions, respiratory and skin manifestation including tightness in the chest, itching and urticaria rash may occur and we will look out for them.

We are considering the use of tablets over syrups as tablets offer advantages over the syrup, which include lower cost, easier dosing, easier ability to comply, and ease of packaging and distribution.

Treatment Chart

| Weight | Age | Dose | Total dose |
| --- | --- | --- | --- |
| 4-9 kg | 6-11 months | ½ tab 3x/day  5ml (1tsp) 3x/day | 4½ tabs  45 ml |
| 10-19 kg | 12 m – 5yrs | 1 tab 3x/day  10ml 3x/day | 9 tabs  90 ml |

**4.11.2 Rationale for choosing Coartem® (artemether - lumefantrine)**

Coartem**®** is currently the first line anti-malarial drug in Zambia for the treatment of uncomplicated malaria. The Zambian MOH has just decided to deploy Coartem® to the community level. It is a fixed combination of two antimalarials, artemether and lumefantrine. It has been proven through clinical trials to be highly efficacious. It eliminates parasites and symptoms significantly faster than most current anti-malarial medications and prevents progression to fatal cerebral malaria. It is rapidly gametocidal, thus helping to reduce transmission while achieving a high cure rate. It’s clinical cure rate is 95% to 97 % in areas with multidrug resistant *falciparum* infection. It is well tolerated showing no evidence of organ or system specific toxicity. It is well tolerated by children (including children between 5 and 10 kg) and can be used in people with renal or hepatic impairment. It will be prescribed according to the manufacturer’s guidelines and in a manner consistent with the Zambian National guidelines. Adverse effects are mostly mild include abdominal pain, anorexia, asthenia, myalgia and arthralgia. In a few cases, children may develop a cough, some pruritis and a rash but none of the adverse events are found to represent a safety problem.

The MOH is planning to procure generic artemether-lumefantrine and when this happens, Coartem® will be replaced with the generic artemether-lumefantrine.

Treatment Chart

| Weight | Age | Dose | Total dose |
| --- | --- | --- | --- |
| 5-14 kg | 1-5 yrs | 1 tab twice a day | 6 tabs |
| 15-24 kg | 5 – 9 yrs | 2 tabs twice a day | 12 tabs |

- - 1. **ICT Malaria Pf as the choice of RDTs**

The ICT Malaria Pf is currently the RDT in use in Zambia. The MOH has procured and distributed ICT Malaria Pf for use in public health facilities. ICT Malaria Pf is a rapid, qualitative two site sandwich immunoassay for the detection of *P. falciparum* specific histidine rich protein -2 (Pf HRP-2) in whole blood. It is both sensitive and specific test for the detection of *P. falciparum* malaria. It is easy to perform and interpret. It has improved stability and the kit can be stored between 4-37°C till the duration of the shelf life. If in the course of the study, the MOH procures another type of RDT, the study will switch to the new type accordingly. There is the possibility that a test can be false positive and therefore a child with malaria may not receive malaria treatment. This condition may worsen. CHWs will counsel caregivers to report back if condition does not improve or worsens.

**4.12 Referral System**

The current referral system is based on verbal referral or at best, a handwritten note. This system will be improved. If during assessment, a CHW decides that a patient needs referral, he will give a start dose of the appropriate medication, if indicated, and will fill a standard referral form which will be in two parts. The CHW will complete the first part, which will provide information on the presenting signs and symptoms (and diagnosis), any treatment given and date and time of referral. He will then give a copy to the patient to be taken to the health facility. At the health facility, the second part of the referral form will be completed when the patient is discharged and given to the caregiver to be sent back to the CHW. This second part will contain information on date and time of arrival to the health facility, date of discharge from the health facility, final diagnosis, and treatment given at the health facility and follow up instructions. When the CHW receives this second part (the “feedback” form), he will update his records with the information and keep the form in a file. During community sensitization activities, NHCs and CPTs will be encouraged to arrange for transportation for referral cases. Referral of sick children to the health center is part of standard care. The research protocol attempts to improve the documentation and feedback

#### 4.13 Monitoring and supervision

Currently, CHWs are visited by RHC staff and the Mobile and Outreach Team from CHS. The visits are supposed to be monthly but are often not this frequent. Some RHC staff instead invite CHWs in their catchment area to the health facility on a regular basis to assist with the work at the facility. During these visits the RHC staff provide support to the CHWs and go through their records. Both methods for providing support to CHWs and supervise their work will be maintained and encouraged in all the study areas. The supervisory visit to CHWs will be linked with the outreach service, which provides immunization, and growth monitoring services to these communities. The DHMT and CHS will facilitate the supervisory visits through the outreach services. During these visits the team will check on the CHW’s routine records of consultations and referrals, as well as medications, supplies and other logistical issues. Restocking of the CHW’s drug supplies will also occur at these visits.

Every three months (the quarterly visit), the health center team will spend a little more time with the CHW in order to undertake some performance assessment.

The NHCs and the HCCs will be encouraged to support and supervise the work of the CHWs. These committees are local structures set up to support community-based agents and health workers for implementation of health programs. They are responsible for initiating and supporting developmental activities and mobilizing communities and local resources to improve household and community health.

#### 4.14 Follow-up

The patients seen by the CHWs with fever and/or fast breathing will be followed up at home by the data collectors to assess the outcome of the treatment given or recommended. The follow-up visit will occur 5-7 days after the visit to the CHW. The data collectors will make contacts with CHWs at least every other day to take particulars of children treated from the identification and baseline forms in order to schedule a follow-up visit. During the follow-up visits, the data collectors will interview the caregiver (the person who took the child to see the CHW or in his/her absence a family member who knows sufficiently about the illness and the care received); and perform some basic examination including taking of temperature and counting of respiratory rate. Information that will be collected during the follow-up visits will include, complaints that prompted heath seeking at the CHW, treatment given, counseling or advice given, referrals, self-reported adherence to the medication, outcome of the visitation, alternative treatment after the visit and where such alternative treatment was obtained and current general condition and complaints. They will also be asked questions to explain inappropriate treatment. If a subject is found not to be improving, the data collector will advise the caregiver to go to the clinic.

#### 4.15 Community mobilization and sensitization

During the initial months of the study, we will conduct a series of meetings with key decision makers and community leaders in the study area as part of efforts to mobilize awareness and support for the study. These community outreach and sensitization meetings will inform the community about the existence and purpose of the study as well as the benefits not only to their communities but also to the country and Africa at large. The reasons for choosing the area and the proposed design will be explained to them. Community leaders will be given the opportunity to decide whether they want their communities to participate in the research. Communities whose leaders oppose to the research will be excluded by not enrolling CHWs working in these communities. Central to this process will be the NHCs, and the chairpersons for each committee. Information will first be disseminated via the chairpersons, and then expand to include local meetings for each committee, and thence to the wider communities that they serve. These activities will also attempt to generate local support for the study and the role the community is expected to play to ensure the success of the study.

#### 4.16 Counseling and Community education

CHWs will provide counseling and basic health information to their patients during consultation. The counseling will include the need to return if condition deteriorates or the child experiences any adverse effects of the treatment. In addition, the CHWs in collaboration with the existing community based health educators in the area: NHCs, CPTs, child survival facilitators; community based distributors; traditional birth attendants and growth monitors will provide basic health information to the communities. Elements of such messages will include:

- General danger signs requiring immediate referral to a health facility
- Symptoms of pneumonia and malaria requiring assistance from the CHW, including danger signs
- Need to use the services of the CHWs
- Importance of adhering to counseling and instructions
- Information about taking the drugs themselves, side effects, need to take with food
- Preventive measures to be used with regard to malaria and pneumonia

#### 4.17 Adverse events

Adverse events from the use of drugs will be tracked in both the intervention and the control arms. Adverse events attributable to the use of RDTs, such as severe bruises, skin infections, and prolonged bleeding following finger pricks will also be tracked in the intervention arm. This will be done at both the CHW and health center levels. Caregivers will be counseled to report any adverse event immediately to the CHW or any nearest health facility. At the CHW, an adverse event form will be filled and the patient referred after which the data supervisor will be informed accordingly. At the health facility level the attending health personnel will manage the patient accordingly, complete the adverse event form and inform the supervisors. The supervisor will report to the Study Coordinator who will review the case and form and report any serious adverse event to the study oversight committee and the data safety monitoring board (DSMB).

#### 4.18 Economic Analyses

The overall goal of the economic evaluation is to determine the cost effectiveness and the difference in economic costs between the use of RDTs in the diagnosis and treatment of malaria (the intervention) and the presumptive treatment of fever with Coartem (the control). The economic analysis will also determine the incremental cost-effectiveness of the use of RDTs in the diagnosis and treatment of malaria so as to provide an additional measure by which to judge the usefulness of the ZIMMAPS intervention. The economic analyses will be based both on cross sectional data collected from health facilities and data collected longitudinally from the caretakers of ill children. The key indicators will be direct economic costs incurred in both the treatment and control areas. We will measure such costs at the household, health care provider, and medical facility levels related to diagnosis and treatment of malaria and pneumonia by CHWs, including referrals. Health outcome will be measured as treatment failure defined earlier.

5. DATA COLLECTION AND MANAGEMENT

5.1 Data Collection

The effects of the intervention package will be collected through four main methods/activities:

#### Routine data collected from CHWs and facility-based records.

#### Performance assessments of CHW skills.

#### Follow-up visits of children treated by CHWs at home.

#### A set of household surveys.

CHWs will keep an outpatient department (OPD) register in which each person attended to will be registered. The OPD register will provide information on the name of the patient, address, sex, age or date of birth, date of onset of illness, main complaints, classification of the disease, drugs given, and comments (recovered, referred or died). The CHW will then make a monthly summary of the register. In addition, the CHW will keep a daily tally sheet for drugs and supplies dispensed and make a monthly summary. The CHW will keep a record of referred cases. The record (which is supplemented by information received from the health center) will include date and time of referral, presenting signs and symptoms (diagnosis), date and time seen at referred centre, date of discharge, and final diagnosis. The CHW will also complete an “identification and baseline form” for every child between six months and five years with fever or fast breathing. The health facilities in the area will keep morbidity and mortality records including information on all CHW referrals.

On a monthly basis the data collectors will visit the CHW and health facility and collect summaries of records kept in special design case report forms (CRFs). The information collected on CRFs for CHWs will include number of children seen, those classified as malaria or pneumonia or both, RDTs performed, drugs dispensed, referrals and whether a supervisory visit was made The information that will be collected on CRF at the health facility will include number of malaria and pneumonia cases, severe malaria and pneumonia cases, referrals from CHWs, deaths by cause, availability of RDTs, Coartem**®** and amoxicillin.

The data collectors will visit the CHWs at least every other day to collect completed identification and baseline forms to schedule a follow-up visit. The DC will check with the CHW register to ensure that every child seen by the CHW with fever of fast breathing has a completed identification and baseline form. The follow-up visits will be made at 5-7 days after the caregiver visits the CHW. Information that will be collected during the Day 5 – 7 follow-up visits will include, complaints that prompted heath seeking at the CHW, whether rapid diagnostic test was done, treatment given, counseling or advice given, self-reported adherence to the medication, outcome of the visitation, alternative treatment after the visit and where such alternative treatment was obtained, current general condition and complaints and results of temperature and respiratory rate taken.

Economic cost data from health facilities will be collected by the data collectors. These data will include the average costs associated with treating uncomplicated and severe cases of malaria and pneumonia, including overhead, equipment, personnel, medicines, and supplies. This information will then be matched with the case records in order to estimate the facility-based costs of treatment for children in intervention and control groups.

Household surveys will be conducted at the beginning (baseline), and at the end of the project to allow us measure some of the outcomes. These surveys will collect information on demography, care seeking behaviors, recent morbidity and mortality, preventive health measures especially for malaria, knowledge of danger signs, adherence to treatment, utilization and acceptability of CHW services, opinion about CHWs. Women resident in the study area aged 15 to 45 years who have at least one child under 5 years will be enrolled for this survey. One member per household will participate. Where there is more than one eligible member, the oldest will be selected. The data collectors will conduct the household surveys. This work will be independent of the follow-up visits. Eligible women will be enrolled based on the inclusion and exclusion criteria irrespective of whether they received care from a CHW or not. Women will be asked about the recent illnesses of their children, including about disease-specific symptoms, whether they sought care, what kind of care they sought, and what kind of treatment they received/followed. The post intervention household survey will also collect information on the acceptability of the intervention.

The CHWs and some opinion leaders in the communities will also be interviewed on their views on the intervention.

- 1. **Supervision of Data Collectors**

Supervision of data collectors will be achieved by assigning 6 DCs to a DS. The DS will visit and work with each of the assigned DC at least once every two weeks. The DS will watch the DC performing part of the assigned job followed by a discussion based on the observations and the DC’s general performance. Any DC whose performance consistently falls below average will be fired and replaced by one of the people on “shelf”.

The supervisors will make three types of supervisory visits

i. Scheduled visits

The Supervisor will visit a home at the same time as a DC, having made an appointment with DC

ii. Random unscheduled visits

The Supervisor will visit a DC unannounced and not by appointment. This visit will provide an opportunity for the supervisor to pick up any “bad” practices that may have cropped up in the course of the field work.

iii. Check visits

The Supervisor will visit a home on his own without the presence of the DC. This visit is performed for several reasons including:

- - - Obtain information from the home whether a reported visit by the DC was made
    - Find out about the conduct of the DC during the visit
    - Discuss any concerns of caregivers taking part in the study
    - Conduct a random blind interview - The supervisor conducts a repeat interview and compares the answers/responses with a similar interview conducted by a DC.

**5.3 Pilot Study**

The data collection instruments, the classification/diagnostic tool and study procedures will be evaluated in a pilot study of at least 2 patients per catchment area. The case report forms, diagnostic tool and study procedures will be modified as necessary based on the results of the pilot study. The data collected during the pilot study will not be used for final analyses and therefore have not been included in the sample size.

**5.4 Data Management**

Data collectors will report to data supervisors. There will be two data supervisors; each will have responsibility for coordinating six data collectors. Each supervisor will make contact with each of the six data collectors he is responsible for, and collect all recently completed case report forms at that time. They will check the forms to ensure that they are completed properly and that there are no blanks. The data supervisors will in turn coordinate with the data manager who will be responsible for double entry of the data into a computer data base. The data manager will run consistency and validations checks and generate errors. The data supervisors will be notified of missing, ambiguous, or non-interpretable data entry on the forms so that these queries can return down the chain back to the responsible data collector for clarification or reconciliation. Once these forms are finalized, primary data analysis will be performed to track accrual and event statistics, and to identify systematic errors that are occurring in the data entry and/or collection process in a timely fashion. The data manager will in turn interface with the chief statistician on the project at BUSPH for the purposes of final data cleaning and analysis.

**5.5 Performance Assessment**

The performance of CHWs will be assessed through two methods on a quarterly basis. The first will be done through the quarterly analysis of the information recorded on the identification and baseline form. From this data, we will be able to determine the CHWs’ performance in classification and treatment. For classification, we will calculate the “percentage of misclassification” (number misclassified based on the complaints out of the total number classified multiply by 100). For treatment, we will calculate the “percentage of inappropriate treatment” (number inappropriately treated based on classification out of the total number classified multiply by 100). Percentage of misclassification or inappropriate treatment of more than ten percent will be considered unacceptable. Such CHW will receive remedial education, which will be arranged by the field coordinator. The second method of assessment will be carried out by the supervisors (rural health center staff or the CHS mobile team) during a normal supervisory and supportive visit. The supervisors will assess the performance of CHWs by observing them attending to patients at the community health post including performance and interpretation of RDTs. If supervisors realize that there are not enough patients for observation at the community level, CHWs may be brought to the health facility and given patients to attend to in order to assess their performance. The supervisor will report the performance as “satisfactory” or “not satisfactory” on the supervisory checklist depending on the CHWs ability to classify and provide the appropriate management or perform and interpret RDTs. This information will be captured on the Health Facility Data Capture Form. CHWs with “unsatisfactory” performance will receive remedial education.

**5.6 Measurement of economic costs**

###### Types of costs measured

The data collectors will obtain economic indicator data to allow us to estimate the total direct costs associated with the intervention, the difference in costs between the intervention and control groups, and the cost-effectiveness of the intervention. This will involve collecting data on two major categories of direct costs, and at different levels, as follows:

1) *Direct medical costs*: those medical costs related to diagnosis and treatment of malaria at the household, CHW and health facility levels, including facility-based overhead/administration, personnel time, bed fees, medications and supplies, diagnostic tests, and outpatient consultations.

2) *Direct non-medical costs*: those costs unrelated to medical care, including transportation, meals, and other expenses associated with diagnosis and treatment seeking, as well as training costs, non-medical supplies and materials, and community education/sensitization activities.

###### Cost measurement

Costs will be estimated on the basis of their resource value. Costs will be estimated using both information provided by caretakers on specific treatment seeking activities and estimates of associated costs at medical facilities. These facilities will include Chikankata hospital, rural health care centers, and CHW health posts. We will estimate the cost of overhead/administration (where not included in the bed fee) with the assistance of facility staff and other available data, as well as the costs associated with facility services, including personnel time, medications, diagnostic tests, and supplies.

The costs of non-facility provided medications, diagnostic tests, supplies and other medical items will be valued at their market cost, as will direct non-medical expenses paid by caregivers such as meals and transportation.

At the program level, the costs associated with implementing the intervention, such as training and materials, will be estimated on the basis of the costs of production and implementation of the intervention. All research-specific costs related to data collection will be excluded, as these are costs of the study and not of providing the intervention to the community. For example, any cost of supplying bicycles or motorized transportation for data collectors is a research-related cost that would not be incurred in actual implementation of the CHW intervention and thus would be excluded from the total cost of this intervention.

**6.0 Statistical Analyses**

# 6.1 Primary end-points comparison

There are two primary endpoints for the study depending upon whether a child has fever or fast breathing. For children with fever the primary outcome is appropriate treatment with Coartem® and for children with fast breathing the primary outcome is early and appropriate treatment. For each primary outcome, crude proportions in the two groups will be compared using the Mantel-Haenszel chi-square test with clusters as strata to account for the cluster randomization. Crude data will be presented as cluster adjusted odds ratios and 95% confidence intervals. Inferences about treatment effects will be made at the level of the individual not at the level of the cluster. Analyses will be based on an intent-to-treat basis.

# Confounding

Because this is a cluster-randomized trial, the potential for confounding is greater than in an individually randomized trial. To adjust for this we will collect data on predictors of inappropriate treatment within clusters. Independent predictors of inappropriate treatment will be used to adjust for confounding in the final regression analysis.

# Multivariable regression

To account for the clustering as well as to adjust for potential confounding, we will adjust the final results using a multivariable regression model. We will use generalizing estimating equations (GEE) ^24^ to estimate the log odds of the intervention on inappropriate treatment proportion compared to the control group. Use of GEE will allow us to adjust variance estimates (providing robust standard errors) to account for the clustering of cases within a cluster. The correlation matrix will be defined as compound symmetry. Odds ratios and 95% confidence intervals will be used to summarize the data.

# 6.2 Secondary end-point comparison

The secondary outcomes are:

1. To measure the proportion of children managed in each study arm who does not respond to treatment (treatment failures).
2. To measure the cost-effectiveness of having CHWs manage presumptive malaria using Coartem® with and without the use of RDTs.
3. To measure the proportion of acute febrile illnesses in children which are RDT-positive.

The analysis of the secondary outcomes 1 and 3 would be similar to the analysis of the primary outcomes. The analysis of secondary outcome 2 is described below.

# 6.3 Analysis of economic outcomes

###### Univariate and multivariable analyses

Analyses will be conducted at the provider level (encompassing both households and facilities). We will calculate the mean costs of diagnosis and treatment of malaria pneumonia for intervention and control groups, their standard deviations, medians, and ranges. Group differences in costs will be assessed using chi-square tests. Univariate analyses will also be conducted for likely predictors of costs, including household socio-demographic characteristics (i.e., education of mother, household size), distance from household to nearest health facility, and treatment history details (i.e., referral to RHC, time from diagnosis to RHC visit, follow-up on referral, hospitalization).

We will conduct multivariate regression to estimate the effect of various factors, including household socio-demographic characteristics and study arm, on the total cost by day 5-7 of treatment of malaria.

###### Estimate of incremental cost per health outcome

Several of our specific secondary objectives will involve calculating cost-effectiveness ratios (CERs). Each CER will be estimated by calculating the difference in costs divided by the differences in health outcome according to the following standard cost-effectiveness formula:

*CER = (C_A_ – C_B_) / O_A_ – O_B_),*

Where C refers to total cost, O refers to outcome, A refers to the intervention group, and B refers to the control group.

###### Sensitivity analysis

We will conduct sensitivity analysis to determine which variables most significantly affect our results. This will be done by univariate and multivariate sensitivity analysis, as well as possibly Monte Carlo Simulation modeling, on our main economic outcome variables.

**7. Informed Consent Process**

As a community wide intervention, consent will be obtained at three levels. At the first level, community leaders and village headmen will be approached through the neighborhood health committees and briefed on the project and their consent and approval will be solicited. Community leaders will be given the opportunity to decide whether they want their communities to participate in the research. Communities whose leaders oppose the research will be excluded by not enrolling CHWs working in these communities. This process will be monitored by the local IRB.

The second level of consent seeking will be from the CHWs. This will be an informed consent. This is because in addition to collecting data on their performance, we also collect personal information including social and demographic data. The field coordinator with assistance from CHS and the Mazabuka and Siavonga DHMTs will visit all the CHWs supported by CHS in the study area. The project will be explained to them stressing their roles and responsibilities and the benefit to the community if the project proves feasible. It will be emphasized that there will not be any payment for their role in the project. It is only when they have signed an informed consent that they will be invited for training. Those who do not consent will not be trained and therefore patients they attend to will not be followed up.

The third level of consent will be from caregivers of children who will be followed up after receiving treatment from the CHWs. The consent is to allow us to collect data by which to assess the effectiveness of the intervention “CHWs delivery Coartem with the aid of RDTs and amoxicillin”, NOT for treatment per se as would be the case in a traditional efficacy trial.

The consent will be taken when the data collector makes the day 5-7 follow-up. It is only when the caregiver has signed or thumb-printed the consent form that the follow-up form will be administered. We will also take consent from respondents of the household surveys before they are interviewed.

The data collectors will take the consent for the follow-up visits and the household surveys. .In all cases the person obtaining the consent will explain the study, including all requirements and benefits to the community. All those consenting will be assured of the confidentiality of the data that will be collected. Those who are willing to participate will be asked to sign the written informed consent, which will be countersigned by the person obtaining the consent. Those who are illiterate will be allowed to thumbprint the consent form instead of providing a signature.

All consent forms will be developed in accordance with guidelines of Boston University’s IRB and the Ethics Committee of the University of Zambia. They will be translated into Tonga and then back-translated into English to ensure validity of their contents.

# 8.0 Data Safety Monitoring Board

We will constitute a data safety monitoring board to conduct an interim analysis at six months on the primary outcomes and review any serious adverse events that may occur in the study and make an independent determination of whether they are study-related or not. The DSMB members will be comprised of two Zambian scientists, possibly a statistician and a pediatrician and one US-based epidemiologist, none of whom will be directly involved in the study. The study will be stopped if during the interim analysis we find that the appropriate treatment for fast breathing is increased by 40% or more and inappropriate use of Coartem for fever is reduced by 40% or more in the intervention arm.

**9.0 POTENTIAL RISKS/DISCOMFORTS AND BENEFITS**

**9.1 Potential Risks/Discomforts to CHWs**

The CHWs' participation in the study may pose additional risk beyond what they would normally experience occupationally. The misuse or the perception of misuse of medication or misclassification could cause their reputation to be tainted or harmed in the community. This will be minimized through regular performance assessment and providing feedback to the CHWs. There is also the risk of pricking themselves during the performance of RDT. This will be minimized by emphasizing the need not to rush and to use extreme care when handling sharp objects and also how to dispose off sharp objects during the training. They will also be taught what to do in the event of them pricking themselves.

**9.2 Potential Risks/Discomforts to patients**

For children in the intervention arm, we anticipate that the research will involve greater than minimal risk, but presents the prospect of direct benefit to society. The subjects are children that have ordinarily reported to CHW for treatment of fever and/or cough/difficult breathing. Even though these children will be treated with proven efficacious and safe drugs which are accepted as first line drugs at public health facilities and will be given per the standard national treatment guidelines, there are some safety issues that may arise. Since the CHWs are not accustomed to prescribing these drugs, there is the possibility of misuse and potentially the occurrence of adverse effects.

There is also the likelihood of misclassification of severe pneumonia cases as pneumonia and therefore treating them at the CHW level instead of referring them to the health facility and these may result in worsening conditions. There may be a few pneumonia cases which fail treatment and become serious cases.

The pricking of the hand for RDTs may hurt for a moment and a small bruise or slight bleeding may appear at the site of the finger prick. There is also a very low risk of soft tissue infection. There is the possibility that a test can be false negative and therefore a child with malaria may not receive malaria treatment. This child’s condition may worsen.

These potential risks will be minimized through training and regular monitoring and supervision of the work of the CHWs. The risk associated with the finger prick will be minimized by the use of sterile techniques and application of pressure at the site of the prick. The health center staff during the monthly supportive and supervisory visits will review cases and referrals. Caregivers will also be advised to report any adverse effects and to return for review immediately if there is deterioration of the child's condition or if there is no improvement 48 hours after seeing the CHW. The CHWs will be informed that if they are not sure whether a case is pneumonia or severe pneumonia, they should take it as severe pneumonia and refer.

**9.3 Potential Risks/Discomforts to participants of the Household surveys.**

The interview may take time to complete and may cause some discomfort. The recall of illnesses and deaths may cause some upset and bring memories of grief and sorrow. The training of interviewers will be aimed at improving their communication and interviewing skills.

**9.4 Potential Benefits to study subjects**

The training the CHWs will receive will improve their skills and may increase their confidence in attending to children. The improved skills of the CHWs are likely to make them provide better quality of care, which will be beneficial to the patients they attend to including the participants of the study.

## 9.5 Potential Benefits to Society

## The results of this study may have an influence on national policy and thus lead to changes that would allow CHWs to use Coartem with RDTs and amoxicillin (efficacious drugs for malaria and pneumonia) at the community level. This will bring early and appropriate treatment of malaria and pneumonia (two of the leading cause of deaths in children) to many children in developing countries. This becomes particularly relevant to areas where this is little possibility of referral or the care taker cannot be referred (distance or other home obligations). This new approach might also result in decrease in the costs of care.

## 10.0 Study Organization and Implementation

## 10.1 Collaborators

## The study will be collaboration between the Center for International Health and Development of Boston University School of Public Health, The Ministry of Health of Zambia and the Chikankata Health Services of the Salvation Army Corp of Zambia. The Ministry of Health will be represented by the Child Health Unit, the National Malaria Control Center and the District Health Management Teams of Mazabuka and Siavonga Districts. The Chikankata Health Services and the DHMTs will be the implementation agencies while CIHD, the Child Health Unit of MOH and NMCC will provide the technical support and assistance. There will be a memorandum of understanding between these partners, which will be passed by the Ministry of Health Headquarters.

## 10.2 Study Coordination

There will be a study coordinator who will be responsible for overseeing implementation of field work and collection of the requisite data in collaboration with implementing partners. He will also be responsible for regular reporting to the BU research team and the Steering Committee, and for maintaining regular communication between the collaborating teams and community leaders. Requirements: ability to manage small field office, some data management skills, language skills, good interpersonal skills.

**10.3 Dissemination:**

We plan to share the findings with the Zambian MOH and other key stakeholders in Zambia through provincial and national dissemination meetings. Manuscripts will also be written for publication in peer review journals.

## 10.4 Study Timeline

The study will take two years to complete, six months for preliminary activities, which will include baseline data collection and formative research, one year of intervention and data collection, and six months of data analysis, reporting and dissemination.

A proposed timetable for ZIMMAPS is presented below.

| Table 1: TIMELINEACTIVITIES | ***Year and Months***  ***2007 2008 2009*** | | | | | | | |
| --- | --- | --- | --- | --- | --- | --- | --- | --- |
|  | 6-8 | 9-11 | 12-2 | 3-5 | 6-8 | 9-11 | **12-2** | **3-5** |
| IRB Approvals | **X** | **X** |  |  |  |  |  |  |
| Community sensitization activities | **X** | **X** |  |  |  |  |  |  |
| Training of CHW |  | **X** |  |  |  |  |  |  |
| Pilot testing of study instruments |  | **X** |  |  |  |  |  |  |
| Finalization of study instruments |  | **X** |  |  |  |  |  |  |
| Implementation of integrated Management and data Collection |  |  | **X** | **X** | **X** | **X** |  |  |
| Interim analysis/DSMB review |  |  |  |  | **X** |  |  |  |
| Preliminary data analyses/abstracts |  |  |  |  |  |  | **X** |  |
| Data analysis workshop (in Zambia) |  |  |  |  |  |  |  | **X** |
| Manuscripts/reports written |  |  |  |  |  |  |  | **X** |

**11.0 References**

(1) Williams BG, Gouws E, Boschi-Pinto C, Bryce J, Dye C. Estimates of worldwide distribution of child deaths from acute respiratory infections. Lancet Infectious Diseases 2002; 2(25):32.

(2) World Health Organization. World Health Report. 2002. Geneva, Switzerland,

World Health Organization.

(3) Snow RW, Craig M, Deichmann U, Marsh K. Estimating mortality, morbidity and disability due to malaria among Africa's non-pregnant population. Bull World Health Organ 1999; 77(8):624-640.

(4) Snow RW, Guerra CA, Noor AM, Myint HY, Hay SI. The global distribution of clinical episodes of *Plasmodium falciparum* malaria. Nature 2005; 434:214-217.

# (5) MOH, NMCP: A 5-year Strategic Plan “A Road Map for RBM Impact in Zambia”

# 2006- 2010

(6) Winch PJ, Gilroy KE, Wolfheim, Starbuck ES, Young MW, Walker LD, Black RE. Intervention models for the management of children with signs of pneumonia or malaria by community health workers. Health Policy & Planning. 20(4):199-212

(7) Schellenberg JA, Victora CG, Mushi A et al. 2003. Inequities among the very poor: health care for children in rural southern Tanzania. The Lancet 361: 561–6.

(8) Victora CG, Wagstaff A, Schellenberg JA et al. 2003. Applying an equity lens to

child health and mortality: more of the same is not enough. The Lancet 362: 233–41.

(9) Armstrong Schellenberg JR, Adam T, Mshinda H et al. 2004b. Effectiveness and

cost of facility-based Integrated Management of Childhood Illness (IMCI) in

Tanzania. The Lancet 364: 1583–94.

(10) El Arifeen S, Blum LS, Hoque DM et al. 2004. Integrated Management of

Childhood Illness (IMCI) in Bangladesh: early findings from a cluster-randomised

study. The Lancet 364: 1595–602.

(11) WHO/UNICEF. 2004b. Joint Statement: Management of Pneumonia in

Community Settings. WHO/FCH/CAH/04.06 or UNICEF/PD/Pneumonia/01.

Geneva and New York: World Health Organization, Department of Child and

Adolescent Health and Development, and United Nations Children’s Fund,

Programme Division.

(12) Pandey MR, Daulaire NM, Starbuck ES, Houston RM, McPherson K. Reduction in

total under-five mortality in western Nepal through community-based antimicrobial

treatment of pneumonia. Lancet 1991; 338:993-997.

(13) Garg R. Omwomo W. Witte JM. Lee LA. Deming MS. Care seeking during fatal

childhood illnesses: Siaya District, Kenya, 1998. American Journal of Public Health

2001; 91 (10): 1611-3

(14) Fauveau V, Stewart MK, Chakraborty J, Khan SA. Impact on mortality of a community-based programme to control acute lower respiratory tract infections. Bulletin of the World Health Organization 1992; 70:109-116.

(15) Sazawal S, Black RE. Effect of pneumonia case management on mortality in neonates, infants, and preschool children: a meta-analysis of community-based trials. Lancet Infectious Diseases 2003; 3:547-556.

(16) Kidane G, Morrow RH. Teaching mothers to provide home treatment of malaria in Tigray, Ethiopia: a randomised trial. Lancet 2000; 356(9229):550-555.

(17) Pagnoni F, Convelbo N, Tiendrebeogo J, Cousens S, Esposito F. A community-based programme to provide prompt and adequate treatment of presumptive malaria in children. Transactions of the Royal Society of Tropical Medicine & Hygiene 2005; 91:512-517.

(18) Moody A. Rapid diagnostic tests for malaria parasites. Clin. Microbiol. Rev.

2002; 15(1): 66-78.

(19) Karin Källander, Jesca Nsungwa-Sabiiti Stefan Peterson. Symptom overlap for

malaria and pneumonia—policy implications for home management strategies. Acta

Tropica 90 (2004) 211–214

(20) Kelly JM, Osamba B, Garg RM et al. 2001. Community health worker performance

in the management of multiple childhood illnesses: Siaya District, Kenya, 1997–

2001. American Journalbof Public Health 91: 1617–24.

(21) Mazabuka District Health Management Team Disease Incidence Report 2005

(22) Savionga District Health management Team Disease Incidence Report 2005

(23) Rennie, W. et al., Minimising human error in malaria rapid diagnosis: clarity of

written instructions and health worker performance, Trans. Roy. Soc. Trop. Hyg.

(2006), doi:10.1016/j.trstmh.2006.03.011.

(24) Peters TJ, Richards SH, et.al. Comparison of methods for analysing cluster

randomized trials: an example involving a factorial design. Int J Epidemiol 2003;

32(5):840-6
