# Supplementary material for: Community Case Management of Fever Due to Malaria and Pneumonia in Children Under Five in Zambia: A Cluster Randomized Controlled Trial
Source: PLoS Med. 2010 Sep 21;7(9):e1000340. doi: 10.1371/journal.pmed.1000340 (PMC2943441; doi:10.1371/journal.pmed.1000340)
Supplement: Text S3 — CHW training manual. (0.31 MB DOC) [file pmed.1000340.s003.doc]

# Community Health Worker

# Training Manual

**Zambia integrated management of malaria and pneumonia STUDY**

# (ZIMMAPS)

# *Adapted from the Zambian Reference Manual for CHWs*

# INTRODUCTION

***What is in this manual?***

This manual is designed to serve as a teaching and reference tool for the community health workers (CHWs) participating in the ZIMMAPS study. It is supplementary to the ZIMMAPS standard operating procedures manual. For the intervention CHWs, this manual will be used in conjunction with the “RDT training manual”. These manuals are intended to provide the CHWs the needed knowledge and skills to perform their roles in ZIMMAPS. It has been organized into six major sections.

Section 1 deals with recognizing and treating malaria

Section 2 deals with identifying and managing children with pneumonia.

Section 3 describes how to use the designed treatment algorithms

Section 4 deals with how to refer a sick child to the health facility

Section 5 deals with managing drugs and supplies

Section 6 deals with keeping the relevant records to be used in the study.

**Section 1**

**Malaria**

Malaria is the most common cause of illness and death among children under *5* years of age in Zambia. Most of the visits and admissions to health facilities are due to malaria. Malaria can be prevented and cured. In this chapter CHWs will learn how to care for children with malaria and to provide appropriate treatment in order to reduce the number of deaths due to malaria.

**Transmission of Malaria**

**How is malaria passed from person to person?**

A person gets malaria when a mosquito that is carrying malaria bites them. There are 5 steps that you need to know about how malaria is carried from one person to another.

*Five steps of malaria transmission*

Step One:

A malaria mosquito lands on a person who is already sick with malaria. The person who is sick with malaria has malaria parasites in their blood. A parasite is a small kind of germ.

Step Two:

The malaria mosquito bites the sick person. When it does, it sucks the sick person’s blood and parasites into its own body.

Step Three:

The parasites grow in the mosquito for 10-14 days.

Step Four:

The malaria mosquito bites a well person. The mosquito injects the parasites from its body into the blood of the well person.

Step Five:

Once in the blood, the malaria parasite multiplies. From the time of the bite, it takes from 7 to 14 days for the person to feel sick from so many parasites in their blood.

***Important facts about malaria mosquitoes and person to person transmission***

- Malaria can occur throughout the year, but it is most common during the rainy season.
- Many mosquitoes come out at night and are bothersome, but not all mosquitoes transmit malaria.
- Mosquitoes that make noise are not malaria mosquitoes. Malaria mosquitoes are silent.
- Malaria mosquitoes come out mainly late at night (20:00hrs) to early in the morning, (02:00hrs). Not as much in the early evening.
- Malaria mosquitoes can fly as far as 7 km away from the community.
- Malaria mosquitoes can breed even in small amounts of water such as a footprint in the rainy season.

**Who are at highest risk of illness and death from malaria?**

The most vulnerable to malaria are:

•  Pregnant women

•  Children under five years of age

•  People with chronic illnesses such as HIV/AIDs

It is important to know who these people are since they need special protection from malaria. Families and communities should take special care to ensure that these people do not get infected with malaria.

**How is malaria prevented?**

Protecting ourselves from mosquito bites prevents malaria. That is the only way we can be sure that we will not get sick from malaria.

Some ways of preventing malaria are better (more effective) than others.

**Methods of protecting against malaria**

- **Sleeping under insecticide treated bed-nets.**
- **House spraying**
- Use repellants. Repellants are special chemicals that help to keep mosquitoes away.
- Wearing the right clothes especially in the night
- Use of mosquito sprays and coils
- Filling in or draining places where water collects and mosquito breed
- Eliminating stagnant water where mosquitoes can grow and clearing areas around the house where mosquitoes can hide during the day.

**Recognizing the signs and symptoms of malaria and treating malaria**

Malaria usually occurs as a sudden illness. Signs of malaria may include:

- Fever
- Feeling cold (shivering)
- Profuse sweating
- Nausea and vomiting
- Convulsions
- Headache
- Painful joints
- General body pains
- Poor appetite
- Restlessness
- Loss of interest in one’s surroundings
- Bitterness of mouth

**The most important symptom/sign of malaria is history of fever or documented fever (temperature ≥37.5°)**

**If there is no history of fever or documented fever (temperature ≥37.5°), then it is not malaria**

**Every fever is not malaria**

**Malaria can co-exist with other causes of fever.**

**Treatment of malaria**

**Treatment of children more than 5kg and above 6 months of age**

Children who weigh more than 5kg and are above 6 months of age who have uncomplicated malaria and no danger signs should be treated with Coartem.

**In the control arm:**

Give Coartem to these children when they have a history of fever **OR** feel hot **OR** have a temperature of 37.5°C or above

**In the intervention arm:**

A. Give Coartem only when the **RDT test is positive**

▪ The child should take the first dose in the presence o of the Community Health Worker.

▪ The Coartem chart below gives you the doses.

| Weight | Age | Dose of Coartem | | |
| --- | --- | --- | --- | --- |
|  |  | Day 1 | Day 2 | Day 3 |
| 5-15 kg | 1-3 yrs | 1^st^ dose: 1 tab  2^nd^ dose: 1 tab after 8 hours | 1 tab twice a day (morning and evening) | 1 tab twice a day  (morning and evening) |
| 15-25 kg | 3 – 5 yrs | 1^st^ dose:2 tabs  2^nd^ dose: 2 tabs after 8 hours | 2 tabs twice a day  (morning and evening) | 2 tabs twice a day  (morning and evening) |

▪ The CHW should teach the caretaker how to give the rest of the Coartem at home preferably with fatty food

▪ If the child vomits within 30 minutes of taking the Coartem, the caretaker should give

another dose and return to the CHW for extra doses.

Advice caretaker to return immediately if:

- The child is unable to drink or breast feed
- Becomes more sick
- Fever is still there 2 days after starting the Coartem.

**Children to be referred to the health facility**

- **weighing less than 5kg or below 6 months of age**
- have signs of complicated or severe malaria
  - convulsions/fits
  - vomiting everything
  - drowsy or unable to awake
  - not able to drink or breast feed
  - passing little urine or dark colored urine
  - yellow eyes
  - very pale
- Still having fever two days after starting Coartem

**RDT negative patients in the Intervention arm:**

- Give analgesics
- Ask patient to return if fever does not resolve in 2 days

# Taking Temperature

# Digital thermometers will be used.

Step 1: Press button to turn on the thermometer

A beep signal will sound and the display will light up

Step 2: Observe the last temperature reading

Step 3. Observe thermometer is ready for use

After 4 second display of the last temperature, the degrees sign display will start flashing.

Step 4. Take temperature by axillary (underarm) method

Point the thermometer upward and place the tip well into the patient's underarm. Fold patient's arm over chest to hold the thermometer in place and keep air away from the underarm. Let the mother hold the child’s arm.

Step 5. Listen to the completion beep feature

Normally, the steady beep will continue for about one minute, then you will hear the **three rapid "completion" beeps**. These three rapid beeps confirm that the temperature measurement is complete and degrees sign will also stop flashing.

Step 6. Read thermometer and record result

Remove thermometer and read temperature on display and record the reading

Step 7. Press button to turn off thermometer

### *Interpretation of results*

Any reading of 37.5°C *or* more indicates the child has a fever.

**Section 2**

**Acute Respiratory Infections (ARI)**

**Cough or cold**

A child that has a runny or blocked nose is said to have a cold. Sometimes the child also has a **cough**. The parents or caretakers may treat a child with a cold or cough at home. A child with cold does not have **fast breathing.**

**Difficult breathing**

“Difficult breathing” means any unusual pattern of breathing. Mothers may use such terms as “noisy”, “fast” or interrupted breathing to describe difficult breathing. Most children with difficult breathing have a cough or cold.

**Pneumonia**

A child with cough or cold or difficult breathing may have a more serious infection called **pneumonia**. When a child has a cough or cold, the caretaker should watch for signs of pneumonia. The signs that will show that the child has developed pneumonia are **fast breathing** and **chest in-drawing**.

Pneumonia is an infection of the lungs. Pneumonia is a cause of death in children and especially young children. A child that has pneumonia should receive medical treatment with antibiotics as quickly as possible.

**You cannot tell the difference between a cough or cold by the nature of the cough itself**.

**A mother reporting that a child has difficult breathing does not necessarily mean that the child has pneumonia.**

**Fast breathing**

When the number of breaths of a child in a minute is the same or more than the cut off breathing rate for the child’s age.

- **One breath includes both breathing in and breathing out**
- **Fast breathing should be determined by counting the respiratory rate and not by the mother telling you.**

A child with pneumonia may have fever (temperature ≥ 37.5°C).

**The presence of the fever does not make it pneumonia.**

**The absence of fever does not make it not pneumonia**

**Chest in-drawing** means that the lower chest wall goes **IN** when the child breathes **IN**. When children have severe chest infection, they require greater effort to breath. As a result, the chest wall moves in. This movement is unusual and is called “chest in-drawing”

The presence of chest in-drawings indicates **severe pneumonia**

# Counting Respiration

# ARI timer will be used

- Be sure the child is calm. If the child is crying or screaming, wait until the child calms down before you start to measure respiratory rate
- Stand where you can see the child’s breathing easily.
- Ask the caretaker to uncover the child just enough for you to be able to see the child’s chest rise and fall.
- Look at the child’s lower part of the chest. This is the part where the last rib can be seen.
- Push the Start/Stop button once.

A short "beep" sound will be heard to show that the timer is starting.

- Actual counting of the respiratory rate must begin as soon this initial beep is heard. Audible clicks will sound every second so that you can be sure that timing is in progress while you continue to observe the patient.
- The timer will give another short beep at the 30 second point of the timing cycle, and two short beeps at the end of 60 seconds when it will automatically shut off. Stop counting when the two short beeps are heard.
- Record the rate and repeat the measurement. It is important to get two measures to be sure that the results are accurate.
- Take the average of the two as the child’s respiratory rate.
- If the difference between the two is more than 5, take a third measurement. Take the average of the nearest two as the respiratory rate.

## Interpretation of the results

1. For young children less than 2 months

Respiratory Rate **≥ 60** Fast breathing (Pneumonia)

1. For children **2 – 11 months**

Respiratory Rate **≥ 50** Fast breathing (Pneumonia)

1. For children **12 months – 5 years**

Respiratory Rate **≥ 40** Fast breathing (Pneumonia)

# Assessing for chest in-drawings

**Chest in-drawings** means that the lower chest wall goes in when the child breathes in. When children have severe chest infection, they require greater effort to breath. As a result, the chest wall moves in. This movement is unusual and is called “chest in-drawing”

- When assessing a child for in-drawing you should disturb the child as little as possible so that he stays calm.
- The mother (and not you) should lift the child’s clothing to expose the chest.
- The whole area of the lower part of the chest moves in, rather than just the spaces between the ribs.
- The inward movement happens whilst the child breathes in.
- The inward movement happens every time that the child breathes.
- The in-drawing occurs even whilst the child is calm.
- If in-drawing cannot be seen clearly, the child should be moved so that he is lying flat in the mother’s lap.
- If in-drawing is still not clearly visible, the child should be classed as not having chest in-drawings.

#### Interpretation of results

The presence of chest in-drawings means the child has severe pneumonia

**Treatment of Cold/Cough**

- Relieve fever with paracetamol
- Clear secretions using clothe soaked in water
- Do not give antibiotics
- Advise mother to
  - Feed the child
  - Return if fast breathing or difficult breathing develops
  - Return if child becomes sicker or is not able to drink or breastfeed

**Treatment for Pneumonia in the Intervention arm**

**Give amoxicillin**

| Weight | Age | Dose of amoxicillin | Duration of treatment |
| --- | --- | --- | --- |
| 4-9 kg | 6-11 months | ½ tab 3x/day | 5 |
| 10-19 kg | 12 m – 5yrs | 1 tab/cap 3x/day | 5 |

- Relieve fever with paracetamol
- Clear secretions using clothe soaked in water
- Advise mother to
  - Feed the child
  - Return if child becomes sicker or is not able to drink or breastfeed

**Treatment for Pneumonia in the Control arm**

Refer to Health facility immediately

Give a referral note

**Treatment for Severe Pneumonia in both Intervention and Control arms**

Refer to Health facility immediately

Give a referral note

**Prevention of chest infections**

| *Prevention of acute respiratory infections*   - Keep young infants warm. Small and ill infants lose heat rapidly especially when wet. The young infants’ feet and hands should be kept warm always. - Avoid unnecessary exposure of all children to cold. To maintain the body temperature, children should always be well dressed. - Promotion of exclusive breastfeeding in children 0 to 6 months of age and continued breastfeeding for 2 years. Breastmilk helps to protect against infections. Children who are breastfed are less likely to become seriously sick and die due to pneumonia. - Immunize all children, especially against measles and whooping cough. Immunizations strengthen the body to fight against infections, including pneumonia. - Avoid indoor air pollution. Children exposed to air pollution are more likely to develop acute respiratory infection. |
| --- |

**Section 3**

**Using the treatment algorithm**

Before using the algorithm, you need to take basic history and perform basic examination

**Basic history**

- Ask for fever and for how long
- Ask for cough/difficult breathing/fast breathing and for how long
- Ask for vomiting. Is the child vomiting everything?
- Ask about feeding. Any difficulty in feeding or drinking?
- Ask for convulsions/fits since the start of the illness
- Ask for diarrhea. Any blood in the stools?
- Ask for any other problems

Basic Examination

- Look for alertness of child. Is he lethargic or unconscious?
- Look for pallor
- Take temperature
- Count respiratory rate
- Look for lower chest in-drawings
- Look for signs of dehydration of there is diarrhea

1. Choose section of the treatment algorithm to use

- Use **Section A** if patients complains of **BOTH fever** and **cough/ breathing problems**
- Use **Section B** if patient complains of **ONLY cough/breathing problems** **WITHOUT** fever
- Use **Section C** if patient complains of **ONLY** fever **WITHOUT** cough/breathing problems

2. Decide whether there is presence of danger signs or not (from history and examination)

- If there is danger signs, Refer immediately

3. In section A or B, Is there fast breathing?

4. In Intervention arm, Is RDT positive or negative?

5. Decide on final action

**Treatment Algorithm for Intervention Arm**

**Treatment algorithm for Control Arm**

**Section 4**

**How to refer a child to a health facility**

**Referral procedures**

There are five steps to take when you refer a child to the health facility.

| *The five steps to take in referring a child:*   1. Explain to the caretaker that the child is very sick and must be taken to the nearest health centre for treatment. 2. Assure the caretaker that the child will receive the best of care/treatment at the health center or hospital. 3. Give clear and specific instructions to caretakers regarding the care of the child on the way. That advice should include the following:    - Keep the young infant warm.    - Clear secretions if nose is blocked.    - Continue breastfeeding the young infant and increase fluids for the older child. 4. Give start dose of appropriate treatment and record on form 5. Write a referral note about the sick child (see example on the Appendix). Give the referral note to the caretaker, who should carry it to the health worker at the health centre. |
| --- |

**Writing a referral Note**

- Date and time of referring
- Your name and address (Name of Community Health Post)
- Name and age of patient
- Main complaints and duration
- Vital signs and significant findings on examination
- Other relevant information:
- Any treatment given
- Sign the form.

**Things to Remember in Referral**

- The Referral form will be a booklet with duplicate
- Must be completed anytime a patient is referred to the Health facility.
- CHW should complete Section A.
- CHW should tear original and give to caregiver
- Health facility staff will complete section B after attending to patient or discharge of patient.
- Patient returns the completed section B back to CHW
- CHW will staple returned half to duplicate.

**Referral note**

Name of CHP___________________________________________

Date:________ /______ *I_____* Time*:______*:*________*

TO: STAFF ON DUTY

_______________________ Health Centre/Hospital.

I am requesting _________________________ Age: ________to come to you for further

investigations and treatment.

He/she was seen here today with the following signs and symptoms:

________________________________________________________________________________________________________________________________________________

________________________________________________________________________________________________________________________________________________

I have given him/her_______________________________________________________

**________________________________**

**Name/**Signature

**………………………………………………………………………………………………Feedback from a health facility**

Name of Health Center/Hospital______________________________________________

Date:_____/_____/_____

The patient: ____________________________Age___________ who was referred

to this health facility for treatment and was seen here on_____/_____/_____(Date) at

_______:______ (Time)

He/She was treated for_____________________________________________________

with____________________________________________________________________

Please continue supervising recovery by:_______________________________________

Return for review on_____ /____ /_____

__________________________________

Name/signature of attending officer

**Section 5**

**Managing Supplies**

Medicines and related supplies, for example test kits and reagents, are expensive and valuable. They need care otherwise they may deteriorate. If they deteriorate, they may lose their potency, have the wrong effects on patients or, in the case of test kits, they may produce incorrect results. Therefore, items in stock should always be stored in a proper storage space.

For this study, we will be managing Coartem, amoxicillin and rapid diagnostic test kits for malaria. CHWs in the intervention arm will manage Coartem, amoxicillin and the rapid diagnostic test kits while the CHWs in the control arm will manage only Coartem.

Managing supplies involves ordering, receiving, storing and issuing. It is important to keep good records of all of the medicines and supplies you receive. This helps you to know:

- What items are available in stock
- How much is available of each item in stock
- How much stock is used on a regular basis
- When and how much of an item should be reordered.

**Keeping records** saves you time and protects you. If you are accused of theft or misuse of supplies, you will be able to refer to your records. Your records will document the movement of supplies. They can show that you are not responsible for the problem.

Stock card and dispensing record

| Date | Opening stock (A) | Received (B) | Issued (C) | Closing stock (D)  D =(A+B)-C |
| --- | --- | --- | --- | --- |
|  |  |  |  |  |
|  |  |  |  |  |
|  |  |  |  |  |
|  |  |  |  |  |
|  |  |  |  |  |

- You will keep a record in a note book
- Keep separate record for each item (5-15 kg Coartem pack and 15-25 kg Coartem pack are considered separate items)
- Make daily entries at the close of the day except you never received nor issue an item to a patient
- Make a summary at the end of each month
- Opening stock on first day of the month, plus total received, minus total issued must be equaled to closing stock at the end of the month.
- Make a physical count at the end of the month to ensure the amount you have in stock equals the balance in your record.
- You will be supplied with the items every month but if you notice that what you have is not enough to last you for the rest of the month THEN you will have to ask for more

**Section 6**

**Keeping Records**

For the purpose of this study, you will keep and maintain two important forms; **the OPD Register** and the **identification and baseline form**.

**The OPD Register.**

- Each person attended to will be entered in this register.
- The OPD register will provide information on the name of the patient, address, sex, age, main complaints, temperature, weight, in-drawings, RDT, diagnosis, treatment, referrals and remarks.
- This is a not a new register being introduced by the study. However, a few items have been added to the existing register.
- You already keep this register and you use information from it to complete the Monthly Aggregation form which you summit monthly to the rural health center or the DHMT.

**The identification and baseline form.**

- This is a new form introduced for the study.
- It will be completed for every patient between 6 months and five years who you diagnose as having fever/malaria and/or fast breathing/pneumonia.
- For the intervention arm it includes patients RDT was performed irrespective of the result.
- This form is designed to record information on presenting complaints, classification, treatment and address of all patients eligible for follow up.
- The form will be filled immediately after attending to the patient.
- The information on this form will be used to schedule Day 5-7 and to locate the home of the patient for the interview.
- This form will also be used to measure correct classification and appropriate treatment.
- The data collector will visit the CHW every other day to collect these forms.

**Remember**

- The data collector will check from the OPD register whether all eligible patients on the register have identification and baseline forms.
- The data collector will also check whether the information on the form is consistent with the information in the Register.

**The CHP Data capture form.**

- This form will not be completed by the CHW but rather the data collector
- It will be completed every month for each community health post
- It will collect data on the activities at the community health post
  - Patients seen
  - Malaria and pneumonia cases seen and treated
  - Referrals
  - Drugs used
