# Supplementary material for: Community Case Management of Fever Due to Malaria and Pneumonia in Children Under Five in Zambia: A Cluster Randomized Controlled Trial
Source: PLoS Med. 2010 Sep 21;7(9):e1000340. doi: 10.1371/journal.pmed.1000340 (PMC2943441; doi:10.1371/journal.pmed.1000340)
Supplement: Text S4 — CHW RDT training manual. (1.93 MB DOC) [file pmed.1000340.s004.doc]

How To Use a ***Rapid* *Diagnostic Test*** (RDT)

A guide for training at the community health post level

*Adapted from a Guide for training at a Village and Clinic level*

Section 1: Introduction

**Learning Objectives**

- Participants will be able to describe a rapid diagnostic test and why the malaria control program has elected to use RDTs in the country/district.
- Participants will be able to describe appropriate actions for RDT positive and negative results.

**Topics to cover**

- What are RDTs?
- Why are they important for malaria control?
- Limitations of RDTs
- Actions for positive and negative RDT results

**What are RDTs?**

Rapid diagnostic tests or RDTs are a way to test whether a person with malaria-like symptoms actually has malaria. Malaria is caused by a parasite that infects blood cells. The parasite is what causes the fever and other symptoms common to malaria. Malaria parasites produce chemicals (proteins) called *antigens.* RDTs detect these malaria antigens in a person’s blood. If malaria antigens are present, the person will test positive. If malaria antigens are not present, the person will test negative. Different types of RDTs detect different antigens. Some antigens are produced by a single species of malaria parasite (e.g. *Plasmodium falciparum*), some are produced by all malaria species (including *P. vivax, P. ovale* and *P. knowlesi*). If present, the antigens cause microscopic particles to stick to a band on the RDT, eventually forming a visible, coloured line in the ‘test’ area.

**Why are RDTs important for malaria control?**

- In the past, most people have used two methods to diagnose malaria:

– The first method is called ‘microscopy’. Microscopy means taking a small amount of blood from the patient and looking at it under a microscope to check for malaria parasites. But many places cannot use microscopy because there is no microscope available or because there is no one trained to find malaria parasites using a microscope.

– The second method is called ‘clinical’ or ‘presumptive’ diagnosis. Clinical/presumptive diagnosis means that health workers do not test to see if the patient has malaria parasites. Instead, they presume that anyone with fever has malaria. The problem with clinical diagnosis is that many different illnesses cause fever and other symptoms common to malaria. Many people with these symptoms do NOT actually have malaria. When everyone with fever is treated for malaria, antimalarial drugs are wasted. Even worse, people with other illnesses do NOT get the right treatment for the illness they have.

- RDTs are a simple and fast way for health workers to test for malaria parasites in a patient’s blood. They are more accurate than presumptive diagnosis and can be used close to the patient’s home. RDTs can also help identify patients who do not have malaria so that these patients can receive correct treatment.
- RDTs give results in about 15 minutes (check product instructions), so a patient with malaria can begin treatment right away. There is no need to wait for microscope results.
- RDTs do not require any expensive or complicated equipment. Most people can learn to use RDTs in just a few hours. Today’s training will be enough for most of you to learn how to diagnose malaria safely and effectively with an RDT.

**What are some limitations of RDTs?**

RDTs are very effective for diagnosing malaria, but there are some things they cannot do:

- RDTs cannot test how many malaria parasites are present in the blood. They can only test whether parasites are present or absent.
- In fact, RDTs do not detect actual *parasites.* As mentioned before, they detect parasite *antigens.* A person who has taken anti-malaria medication within the last two weeks will test *positive* for malaria with these RDTs even if he or she no longer has parasites.
- RDTs can be damaged by heat and humidity, so an RDT should not be removed from its sealed packet until right before you are ready to use it. If a package has been open for some time before the RDT is used, the RDT may be damaged by heat or humidity and give an invalid (false) result. You should discard this package and use another, unopened, package.
- To work properly, RDTs need blood and a chemical called ‘buffer’. Adding too much or too little blood or buffer can cause the test to give an invalid result or be difficult to read. Adding blood and buffer in the wrong place can also cause an invalid result. This training will show you how much blood and buffer to use and where to add them. You will practice using RDTs to test one another for malaria. Pay careful attention during the training, and use the job aid during the practice session, so your tests give correct results.

**Section 2: How to use an RDT**

**Learning Objective:**

- Participants will have a general understanding of how a malaria rapid diagnostic test is performed.

**Activities to cover:**

- Perform the test on a volunteer with all participants watching;
- While performing the test, explain in detail how to carry out each test step;
- Use the job aid as a visual aid for describing and explaining each test step.

**Specific steps:**

1. Assemble all the supplies you will need, including:

test packet

alcohol swab

sterile lancet

examination gloves

Buffer

A watch or clock to use as a timer

1. Place all these supplies on a table where they will be visible to all participants.
2. Check for the expiry date. (importance of expiry date)
3. Put on a new pair of examination gloves.

Protect self and patient from possible infection with blood-borne diseases, including HIV-AIDS.

5. Open the test packet and remove the contents (Explain how it is used).

- The blood transfer device (loop, capillary tube, pipette, or other) is used to collect blood and transfer it to the test cassette.
- The desiccant sachet protects the test from humidity before the packet is opened. (discard once packet is opened)
- The test cassette is used to conduct the test.
  - Explain the holes and the markings and what each one means:
  - The square hole with the letter “A” is where you add the blood.
  - The round hole with the letter “B” is where you add the buffer.
  - The long rectangular hole (‘window’) separated into two sections is where you read the test results. The larger section with the letter ‘T’ is where you determine the diagnosis. If a red line appears in this section, it means the patient has malaria. If no line appears in this section, it means that malaria was not detected in the patient. The smaller section with the letter ‘C’ tells you whether the test is working correctly. A red line *must* appear in this section for the test to be valid. If no line appears in this section, the test is not working properly and the results are *invalid.* If no line appears in this section, you must discard the cassette and test the patient again using a *new test packet* that has not been previously opened.

6. Write the patient’s name on the cassette. (Explain why)

7. Clean the patient’s 4th finger with alcohol swab (Explain why ).

Least used

Least inconvenience if finger becomes sore

Less likely to be infected

Skin may be thinner

8. Allow cleaned finger to *air dry. (Don’t blow or wipe finger)*

9. Open lancet and prick, preferably towards the side of the pulp (ball) of the finger. (Pricking the midline or tip is more painful).

10. Check to be sure the finger-prick will produce enough blood, then discard the lancet in the sharps container.

- Discard the lancet in an appropriate sharps container *immediately* after using it.
- *Never* set the lancet down before discarding it.
- *Never* discard the lancet in a non-sharps container.
- *Never* use a lancet on more than one person.

11. Collect the droplet of blood using the blood collection device

12. Ensure a good-sized drop is on the finger before collecting.

13. Collect just to the mark. Do not lift the tip of the pipette or tube as this will allow air bubbles to enter.

14. Add the drop of blood to the lower well (hole). Do not deposit blood on the plastic edges of the well.

15. Discard the blood collection device after use.

16. Add exactly the correct number of drops of buffer. Hold the bottle vertically.

17. Wait the correct time (e.g., *15 minutes*) after adding buffer before reading test results.

18. Remove and discard your gloves at this time.

**Section 3: Reading and interpreting test results**

**Learning Objective:**

Participants will gain proficiency at correctly interpreting the different possible RDT outcomes.

**Activities to cover:**

- Explain the three possible test results (positive, negative, invalid) using the job aid as a guide;
- Interpret the results of their tests.

**Tests Results**

- **Line** at position **‘C’ AND** **line** at position **‘T’** = ***POSITIVE.***

- If **lines** appear at both positions, the result is ***POSITIVE*** even if the **line** in position **‘T’** is very ***FAINT.***

- **Line** at position **‘C’** and **NO LINE** at position **‘T’** = ***NEGATIVE***

- **No line** at position **‘C’** AND **line** at position **‘T’** = ***INVALID***

- **No line** at position **‘C’** and **NO LINE** at position **‘T’** = ***INVALID***.

**Interpretation of Results**

POSITIVE results = Treat for malaria: Give Coartem

NEGATIVE results = Do not treat for malaria: Do not give Coartem

INVALID results = Repeat Test.

**Disposal of Waste materials**

You will be given two containers for disposal of the waste products

Sharp objects

Sharp objects, the lancets should be discarded into the Safety box

The safety box will be collected from you for disposal when it is full

Other waste

Other waste materials, like the swab, the cotton wool, the cassette and pipette, the gloves and other materials must be discarded in disposal bucket which will be provided. Empty the bucket after the day’s work.
